# Improving the Health-Benefits of Kales (*Brassica oleracea* L. var. *acephala* DC) through the Application of Controlled Abiotic Stresses: A Review

**DOI:** 10.3390/plants10122629

**Published:** 2021-11-29

**Authors:** Erika Ortega-Hernández, Marilena Antunes-Ricardo, Daniel A. Jacobo-Velázquez

**Affiliations:** 1 Tecnologico de Monterrey, Escuela de Ingenieria y Ciencias, Av. Eugenio Garza Sada 2501 Sur, Monterrey, Nuevo León C.P. 64849, Mexico; erika.orhe@gmail.com; 2 Tecnologico de Monterrey, Escuela de Ingenieria y Ciencias, Av. General Ramón Corona 2514, Nuevo México, Zapopan, Jalisco C.P. 45138, Mexico

**Keywords:** kale, controlled abiotic stresses, health-promoting properties, superfood, secondary metabolism, glucosinolates, phenolic compounds, carotenoids, isothiocyanates, nutraceuticals

## Abstract

Kale (*Brassica oleracea* L. var. *acephala* DC) is a popular cruciferous vegetable originating from Central Asia, and is well known for its abundant bioactive compounds. This review discusses the main kale phytochemicals and emphasizes molecules of nutraceutical interest, including phenolics, carotenoids, and glucosinolates. The preventive and therapeutic properties of kale against chronic and degenerative diseases are highlighted according to the most recent in vitro, in vivo, and clinical studies reported. Likewise, it is well known that the application of controlled abiotic stresses can be used as an effective tool to increase the content of phytochemicals with health-promoting properties. In this context, the effect of different abiotic stresses (saline, exogenous phytohormones, drought, temperature, and radiation) on the accumulation of secondary metabolites in kale is also presented. The information reviewed in this article can be used as a starting point to further validate through bioassays the effects of abiotically stressed kale on the prevention and treatment of chronic and degenerative diseases.

## 1. Introduction

Numerous studies have reported that diets high in vegetables are highly correlated with a reduced risk of developing common chronic diseases [1]. A vegetable that is often on the list of the healthiest foods is kale. Originating from eastern Turkey, kale is one of the oldest leafy green vegetables. During the first millennium, it arrived in Europe, where it settled in various cultures. It was not until the early 1980s that kale became popular in America [2]. Due to the adequate tolerance to unfavorable weather conditions and inexpensive production cost, kale has become an important crop for the agriculture-based economy [3].

Kale belongs to the Brassicaceae family, which also includes cauliflower, broccoli, arugula, and brussel sprouts. It is characterized by a sweet, slightly bitter taste and an appearance similar to a mix of lettuce and Swiss chard. Kale exhibits multiple varieties mainly differentiated by color shades, size, and leaf type. Different shapes of the plant are available, including tree kale, marrow kale, thousand-headed kale and collard. The varieties most commonly grown are the Scotch and Siberian kales [2].

Kale has been widely used worldwide in traditional medicine to prevent and treat different health disorders, including gastric ulcers, high cholesterol levels, hyperglycemic, rheumatism, and hepatic diseases [4,5]. Its health-related benefits have been attributed to a great combination of bioactive phytochemicals, including glucosinolates, carotenoids, and phenolic compounds [6,7]. Likewise, kale has a higher nutritional value compared to other foods. According to the USDA database, 100 grams of raw kale provide 2.9 g of protein, 4.4 g of carbohydrates, 4.1 g of fiber, and only 1.49 g of lipids. In addition, it offers more iron (1.6 mg/100 g) than meat, 2–3 times more calcium (254 mg/100 g) than milk, 3–4 times more folic acid (241 ug/100 g) than eggs, and two times more vitamin C (93.4 mg/100 g) than oranges [3].

The content of primary and secondary metabolites can be modified by diverse factors, including the development stage, harvest season, environmental conditions, postharvest handling, and variety [8]. The manipulation of these metabolites can be used to control levels of desirable compounds and improve plant quality. It has been shown that it is possible to increase the concentration of phytochemicals in other vegetables, such as carrots and broccoli, through the application of abiotic stress [9,10].

Because of the importance of secondary metabolites diversity in kale to treat different therapeutic targets, and the need to standardize the natural chemical variation, a better understanding of the factors affecting their biosynthesis is needed. In this review, the main kale phytochemicals are discussed by putting a special emphasis on molecules of nutraceutical interest, including phenolic compounds, carotenoids, glucosinolates and their derivatives. The pharmaceutical activities, in particular those related with the prevention and treatment of chronic diseases, are highlighted in view of the most recent literature data. Abiotic stresses to enhance the production and accumulation of kale secondary metabolites are presented. Specifically, five systems are reviewed: saline stress, phytohormone application, drought, temperature, and radiation. The information presented in this review is based on recent literature (2016–2021) found on Google Scholar using the following combination of key words: kale + nutraceuticals, kale + abiotic stress, kale + carotenoids, kale + abiotic stresses + secondary metabolites, kale + saline stress, kale + phytohormone, kale + drought stress, kale + temperature stress, kale + ultraviolet radiation, and kale + health promoting properties.

## 2. Kale as a Novel Source of Nutraceuticals

In this section, the main nutraceuticals present in kale and their health benefits are presented. Nutraceuticals are chemical compounds present in foods that exert pharmacological activity, resulting in the prevention and treatment of chronic diseases [11]. Glucosinolates, carotenoids, and phenolic compounds from kale are health-related secondary metabolites associated with several beneficial characteristics, showing various pharmacological effects correlated to their antioxidant activity [12,13]. The main biological activities related to kale are antioxidant, anti-cancerogenic, and protective effects on the cardiovascular and gastrointestinal tract [4,12,14,15].

The antioxidant properties of kale have been previously evaluated through in vivo studies. For instance, Sikora and Bodziarczyk [16] reported lower lipid oxidation products (LOP) and malondialdehyde (MDA) in the blood serum of rats fed with a modified diet with raw and lyophilized kale. Likewise, Horst et al. [17] found that kale extract supplementation in Wistar rats (4 ± 0.2 µg/g) showed protection against H_2_O_2_-induced DNA damage. Moreover, Chung et al. [18] described that treatment with kale juice powder for 8 weeks improved serum lipid profiles by increasing the HDL level and decreasing the triglycerides level in Sprague Dawley rats previously fed with a high cholesterol diet.

The neuroprotective potential of kale has been shown on neuroinflammation mechanisms. The evidence indicates that an extract from Tuscan black kale sprouts reduces inflammatory key-markers (p-selectin, GFAP, Iba-1, ERK1/2, and TNF-α) during cerebral ischemia and reperfusion in rats [19]. On the other hand, kale is also able to block the inflammatory response in the digestive system. Lima de Albuquerque et al. [20] proved that administration of lyophilized kale (500 mg/kg) modulates the colonic microbiota in rats with colitis induced by Trinitrobenzenesulfonic (TNBS) acid. In addition, kale showed intestinal anti-inflammatory effects by decreasing the production of the TNFα and IL-1β, and the MPO activity.

Previous clinical studies focused on evaluating the nutraceutical potential of kale have reported that the consumption of its powder for 8 weeks restored blood pressure and glucose levels within the normal range in subjects with potential metabolic syndrome [21]. Similarly, Kondo et al. [22] showed that intake of kale-containing food at a dose of 7 g and 14 g decreased postprandial plasma glucose levels in healthy Japanese subjects. In addition, the supplementation with kale juice improved serum lipid profiles and antioxidant systems in male subjects with hyperlipidemia [23].

### 2.1. Glucosinolates

Glucosinolates are sulfur- and nitrogen-containing thioglucosides derived from glucose and amino acids. They are found in certain families of dicotyledonous plants, including the *Moringaceae*, *Capparidaceae*, *Resedaceae*, and *Brassicaceae* [24].

**Figure 1:**
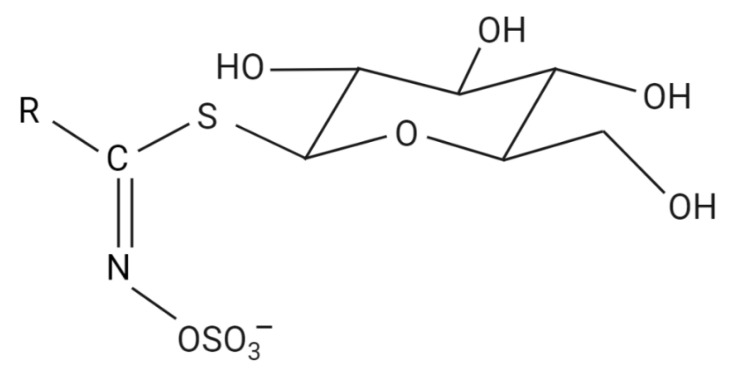
Basic chemical structure of glucosinolates. The basic structure of glucosinolates comprises a thioglucose residue and a sulfate group bound to a central carbon, along with a variable aglycone. Figure created with BioRender.com (accessed on 20 October 2021).

The basic chemical structure of glucosinolates consists of a β-d-thioglucose group linked to a sulfonated aldoxime moiety and a variable aglycone side derived from one of eight amino acids (Figure 1). They are currently classified into subgroups based on their precursor amino acid into aliphatic (alanine, leucine, isoleucine, methionine, or valine), aromatic (phenylalanine or tyrosine), and indole glucosinolates (tryptophan) [25].

Glucosinolates are hydrolyzed by the action of the endogenous enzyme myrosinase (β-thioglucosidase glucohydrolase, EC 3.2.3.1) when the plant tissue is disrupted. Then, the enzyme myrosinase binds with the substrate and releases a molecule of glucose, bisulfate, and the corresponding aglycone. As a result, biologically active compounds are obtained, including isothiocyanates (ITC), nitriles, and thiocyanates, depending on the nature of the aglycone and the physicochemical conditions of the medium (pH, cofactors, and specifier proteins) [26,27]. These molecules contribute to the defense of plants against insects and pathogens and have beneficial properties for human health due to their potential protective mechanisms, including xenobiotic detoxification by induction of phase II enzymes, decreased carcinogen activation by inhibition of phase I enzymes and slowed tumor growth, and induction of apoptosis [6,28,29].

Today, the chemical structure of around 200 different glucosinolates has been elucidated. The qualitative and quantitative glucosinolate profile varies from one species to another, and it is directly dependent on the type of plant tissue. Kale contains glucosinolates in a concentration of 2.25–93.90 μmol/g dry weight (DW basis), but the ratio of indole and aliphatic glucosinolates differs in samples depending on multiple factors, including plant variety, origin tissue, maturity stage, growing conditions, and method of analysis [30].

The predominant glucosinolates found in kale are: glucoerucin, glucoraphanin, progoitrin, gluconapin, glucoiberin, glucobrassicanapin (aliphatics); gluconasturtiin (aromatic); glucobrassicin, 1-hydroxy-3-indoylmethyl, neoglucobrassicin, 4-hydroxy-glucobrassicin, and 4-methoxy-glucobrassicin (indolics) [25,31,32]. Hahn et al. [33] evaluated the glucosinolate concentration and profile in 25 cultivars of 5-month-old kale. The gluconapin content was significantly higher (19.6 mg/100 g FW) in the American varieties (Georgia Southern, Champion, and Vates). In addition, the American kale had significantly more progoitrin than the others. Conversely, American varieties rarely contained glucoraphanin. It was mainly detected in the Italian Black Tuscany (68.69 mg/100 g FW). In most samples, gluconasturtiin was contained in very low amounts, ranging from 0.002 mg/100 g FW (several varieties) to 1.26 mg/100 g FW in Siberian cultivar. Finally, the glucosinolate found in all varieties was glucobrassicin. The highest total amount was detected in the German cultivar Neuefehn (195 mg/100 g FW).

#### Glucosinolates as Bioactive Compounds

The beneficial effects of Brassica vegetables have been attributed to the physiological properties of the glucosinolate breakdown products, ITC. The hydrolysis products of glucosinolates present in kale are associated with numerous therapeutic benefits, summarized in Table 1 and include the potential to reduce the risk of various types of cancers, diabetes, atherosclerosis, and inflammatory and cardiovascular diseases [34,35]. 

ITCs are potent stimulators of enzymes involved in carcinogen detoxification, such as glutathione-S-transferase, which helps neutralize potential carcinogens by turning them into water-soluble compounds and excreting them through urine [36].

**Figure 2:**
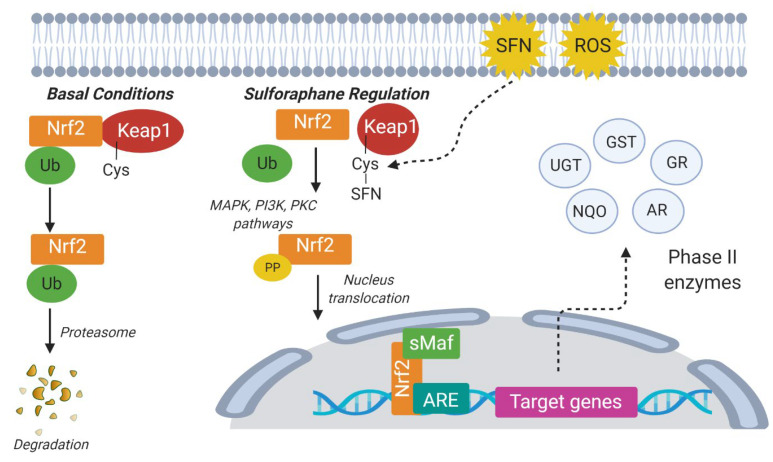
Regulatory mechanism of sulforaphane Keap1-Nrf2-ARE signaling pathway and induction phase II metabolic enzyme expression. Under normal circumstances, Nrf2 binds to Keap1 in the cytoplasm and undergoes ubiquitin-mediated degradation. Under SFN treatment or ROS attack, Nrf2 is activated though the MAPK, PIK3, and PKC signaling pathways, and translocated to the nucleus, where it binds to the promoter ARE region of the target genes and activates the expression of phase II metabolic enzymes. ARE: Antioxidant response element; MAPK: Mitogen-activated protein kinase; PI3K: Phosphatidylinositol 3-kinase; PKC: Protein kinase C; ROS: reactive oxygen species; SFN: Sulforaphane; sMaf: small Maf transcription factors; GST: Glutathione S-transferase; GR: Glutathione reductase; AR: Aldehyde reductase; UGT: Uridine 5′-diphospho (UDP)-glucuronosyltransferase; NQO: NAD[P]H:quinone oxidoreductase [38]. Figure created with BioRender.com (accessed on 20 October 2021).

Stimulation of transcription of phase II enzyme genes by isothiocyanates is achieved through the Keap1-Nrf2-ARE pathway, in which isothiocyanates first bind to thiol residues of the Kelch-like ECH-associated protein 1 (Keap1), leading to conformational changes and eliminating its ability to target NF-E2-related factor 2 (Nrf2) for ubiquitination and degradation. Consequently, Nrf2 translocates into the nucleus, where it forms heterodimers with small Maf transcription factors, binds to antioxidant response elements (AREs) in the upstream promoter region of genes encoding for phase II enzymes, and then accelerates their transcription (Figure 2) [37,38].

Additionally, ITCs can inhibit phase I enzymes, such as cytochrome P450, which are involved in the metabolic activation of most carcinogens in humans [36]. ITCs also effectively induce cell cycle arrest and apoptosis of cancer cells, suggesting their use in chemopreventive treatment [39,40].

Numerous in vitro and in vivo studies suggest that sulforaphane (SFN), indol-3-carbinol (I3C), and iberin may help to reduce the risk of estrogen-sensitive cancer as well as other types of cancer, such as prostate, liver, colorectal, melanoma, and pancreas [41,42,43,44,45,46]. Likewise, Nrf2 is recognized as a significant regulator in oxidative stress and inflammation processes triggered during obesity. All elements of metabolic syndrome are related to the decontrolling of the PI3K/AKT/mTOR, MAPK/EKR/JNK, and Nrf2 signaling pathways [46].

Previous reports have shown that supplementation with glucoraphanin can moderate weight gain, reduce fat storage, and improve glucose tolerance and insulin sensitivity through the activation of the Nrf2 pathway in mice [47]. Similarly, it has been reported that isothiocyanates and indoles, such as sulforaphane and brassinin, suppressed inflammation and inhibited adipocyte differentiation in 3T3-L1 cells and mice through the activation of apoptosis via the ERK pathway [48,49,50,51,52].

Choi et al. [53] described that a treatment with sulforaphane reduced the level of leptin and cholesterol and increased the level of adiponectin in plasma through inhibition of C/EBPα and PPARγ, and activation of the AMPKa pathway. Chuang et al. [54] found that benzyl isothiocyanate and phenethyl isothiocyanate had similar effects diminishing adipogenesis and preventing body weight gain through the inhibition of transcription factors PPARγ and LXRα, and cell cycle arrest at the G0/G1 phase in 3T3-L1 cells. Alike, results for allyl isothiocyanate in literature have showed decrease of body weight gain, diminished fat storing in liver, and reduced inflammation involving the activation of Sirt/AMPKa signaling, the upregulation of PPARα, and the decrease of TNFα, IL1β, and IL6 levels [55]. Besides, benzyl isothiocyanate, phenethyl isothiocyanate, and allyl isothiocyanate reduced hyperglycemia improving insulin sensibility.

Clinical studies directed to evaluate the effect of ITCs and indoles on metabolic syndrome are limited. Bahadoran et al. [56] reported a significant decrease in serum insulin concentration with improved insulin resistance in diabetic patients treated with 10 g/d of broccoli sprouts powder for 4 weeks. Likewise, administration of 30 g/d of broccoli sprouts to healthy overweight individuals for 10 weeks, showed a positive effect on inflammatory parameters with a significant decrease in IL-6 levels [57]. Finally, Kikuchi et al. [58] have reported that healthy men treated with 10 g of nasturtium leaf showed significantly increased levels of peptide YY (PYY), a satiety gut hormone, after intake during 6 h.

Therefore, this evidence supports that the use of glucosinolates and their derivatives from cruciferous vegetables might be considered for the treatment of metabolic syndrome. Human studies have found no significant side effects derived from ITC consumption [59]. Table 2 summarizes the main health benefits of ITC found in kale.

### 2.2. Phenolic Compounds

Phenolics, the most abundant secondary metabolites in plants, are characterized by a common chemical structure, having an aromatic ring with at least one hydroxyl substituent. Based on their chemical structures, phenolic compounds can be classified as flavonoids, phenolic acids, tannins, stilbenes, and lignans [67].

In nature, most phenolic compounds are found glycosylated, although they can also be found esterified or as polymers. Glycosylation of phenolic compounds increases their solubility and stability in water, protecting them from oxidation [68]. Once ingested, the absorption rate, the nature of the circulating metabolites, and their elimination will be determined by their chemical structure [69]. Phenolic compounds are highly relevant in the dietary supplement and pharmacological industries due to their physiological action and health-protective mechanisms [70].

The main phenolic compounds found in kale are hydroxycinnamic acids and flavonoid glycosides, including quercetin, kaempferol, derivatives of caffeic, ferulic, and sinapic acids [6,71,72]. Few comparative studies have been carried out to characterize the main phenolics in kale from different cultivars and origins. So far, anthocyanins have been identified in the red variety of curly kale, predominating cyanidin glycosides [73]. Ferioli et al. [74] compared the phenolic content of kale populations from Italy, Portugal, and Turkey. Portuguese kales showed the highest amount, followed by Turkish and Italian samples. Flavonols were more abundant than hydroxycinnamic acids, accounting for over 80% of phenolics in all samples.

#### Phenolic Compounds as Bioactive Compounds

Phenolic compounds are efficient antioxidants. Proven biological effects of phenolics include inhibiting oxidative effects on proteins, DNA, and lipids by stabilizing free radicals [75]. In this tenor, phenolic compounds exhibit a wide range of beneficial properties for human health, such as anti-allergenic, anti-inflammatory, antimicrobial, anti-carcinogenic, protective effects against cardiovascular and neurological diseases, and have shown the ability to induce vasodilatory effects [76,77,78,79,80,81,82]. Table 3 summarizes the proven health benefits of the main phenolic compounds found in kale.

The neuroprotective potential of flavonoids has been demonstrated through two main mechanisms: oxidative stress and neuroinflammation. The evidence indicates that flavonoids can maintain the integrity and functionality of neurons, and prevent the increase in the production of reactive oxygen species (ROS) and lipid peroxidation in the hippocampus of rats [83]. Improvement on learning and memory in mice, and inhibition of lipid peroxidation and scavenging radicals in neuronal cells due to antioxidant activity of quercetin was reported by Li et al. [84]. In addition, it has been reported that quercetin supplementation in cortical cells culture inhibited up to 61% of the neurotoxicity produced by the addition of *N*-methyl-d-aspartate (NMDA) neutralizing free radicals in brain injury [77]. Flavonoids can also block the inflammatory response related to Alzheimer’s disease by inhibiting microglia, the activity of astrocytes, and pro-inflammatory molecules, such as IL-1β and TNF-α [85]. Flavonoids are steroidogenesis modulators in hormone-dependent cancer, as these compounds can bind to estrogen receptors and DNA [86]. They can also chelate metal ions, such as Fe^3+^, Cu^2+^, Zn^2+^, catalyze the transport of electrons, and neutralize free radicals [87].

Likewise, it has been shown that phenolics protect vitamin E from photooxidation in the cell membrane and inhibit the oxidation of low-density lipoproteins (LDL), thus preventing the formation of atheroma and reducing the cytotoxicity of LDL [88]. Steffen et al. [89] provided evidence that a large variety of flavonoids and their metabolites can protect vascular endothelial cells against O^2·–^, mainly through the inhibition of NADPH oxidase [80]. Also, Perez et al. [78] demonstrated the vasodilator effects of quercetin in healthy volunteers treated with 200–400 mg for 3 weeks. An increase in brachial arterial diameter was correlated with an increase of plasma and urinary levels of glutathione.

Phenolic compounds also have anti-inflammatory and anti-carcinogenic effects related to their radical scavenging activity and their ability to inhibit lipid peroxidation. It has been reported that kaempferol and quercetin have the anti-inflammatory capacity to inhibit the lipopolysaccharide (LPS)-induced PGE2 production, LPS-induced COX-2 expression, and mPGES-1 expression in activated macrophages [79]. Also, kaempferol and quercetin display therapeutic potential as an anticancer drug. Zhang et al. [80] reported that kaempferol and quercetin (50 and–100 μM) act as topoisomerase inhibitors and interrupt the process of DNA replication in HepG2 cells.

Sinapic acid and its derivatives can inhibit NF-κB, which regulates inflammatory status and plays a key role in the immune response to infection. Via NF-κB inactivation, sinapic acid suppresses the expression of proinflammatory mediators, such as inducible nitric oxide synthase, cyclooxygenase-2, TNF-α, and interleukin-1β. Sinapic acid and derivatives have shown antiproliferative action on breast cancer cell lines. Due to its metal-chelating ability, sinapic acid has exerted a protective effect against arsenic-induced toxicity in rats [90].

Ferulic acid and its derivatives are other hydroxycinnamic acids found in kale. These compounds possess several health-promoting properties, most of them related to the treatment of metabolic syndrome, including antioxidant, anti-inflammatory, anti-lipidemic, antidiabetic, antihypertensive, and antimicrobial activity. Ferulic acid also exhibit anti-viral properties as, for instance, Ayaz et al. [82] showed that kale extract rich in ferulic acid inhibits the replication of Gram-positive (*S. aureus*, *E. faecalis*, *B. subtilis*), Gram-negative (*M. catarrhalis*) bacteria, and two yeast-like fungi (*C. tropicali* and *C. albicans*).

### 2.3. Carotenoids

Carotenoids represent a group of more than 600 fat-soluble pigments; they are responsible for the yellow, orange, and red coloration in fruits, roots, flowers, fish, invertebrates, birds, algae, bacteria, and yeasts. Their coloration is due to the high number of conjugated double bonds present in their chemical structure [91]. Carotenoids are generally divided into two classes: carotenes, which are unsaturated C40 hydrocarbons, and xanthophylls, which are oxygenated derivatives of carotenes [91].

In their natural state, carotenoids are bound non-covalently to protein or esterified with saturated fatty acids. Once carotenoids are released from the dietary matrix, they are circulated in the gastrointestinal tract with the help of dietary lipids and bile salts and are associated with lipoproteins in the form of micelles [92]. Xanthophyll esters are hydrolyzed by lipase or esterase and absorbed [93]. A part of provitamin A carotenoids are converted into retinal in the mucous of the small intestine by *β*-carotene-15,15′-dioxygenase. Absorbed carotenoids are incorporated into chylomicrons and then transported to the liver and various organs through the blood. All three major lipoproteins: very low-density lipoprotein (VLDL), low-density lipoprotein (LDL), and high-density lipoprotein (HDL), are involved in the transport of carotenoids [91].

Kale is an excellent source of β-carotene, α-carotene, and lutein [94,95]. Previous reports have shown that kale contains a comparatively higher amount of β-carotene than broccoli, cabbage, cauliflower, and brussel sprouts [3]. The highest levels of β-carotene and lutein content in kale are reached between the first and third week of growth [96].

Kim et al. [97] evaluated the carotenoid concentration and profile in eight common kale cultivars at commercial maturity: Starbor, Beira, Scarlet, Premier, Olympic Red, Toscano, Dwarf Siberian, and Red Russian. Toscano kale was the highest in total carotenoid content due to the high levels of neoxanthin (177.4 µg/g), lutein (712.2 µg/g), and *β*-carotene (958.9 µg/g).

#### Carotenoids as Bioactive Compounds

In plants, carotenoids evolve to serve in photoprotection, oxidative stress, and developmental regulations. In human health, β-carotene and lutein have shown antioxidant and immunomodulation activities, which may prevent degenerative diseases, such as cardiovascular diseases, UV-induced skin damage, cataracts and macular degeneration, diabetes, and several types of cancer, especially prostate and digestive tract tumors [98,99,100,101,102,103,104,105].

The distribution of carotenoids in human organs shows specificity. Lutein is found on the surface of the skin and subcutaneous tissue in an esterified form and acts as a UV absorber and quencher of singlet oxygen [93]. In the eye, lutein and retiral derived from β-carotene are present as macular pigments, acting as light screening and playing an essential role in photoprotection [92]. Ma et al. [98] reported that lutein supplementation (6mg/d or 12 mg/d) to healthy subjects for 12 weeks improved visual function, particularly in contrast sensitivity. Arnold et al. [106] evaluated the use of an oil-based kale extract to improve the vision of AMD-patients. The concentrations of the xanthophylls in plasma and the optical density of the macular pigment increased significantly in the kale group after 4 weeks of intervention.

In addition, carotenoids show cancer-preventive effects through multiple mechanisms. It has been reported that carotenoids cause cell cycle arrest and induce apoptosis and differentiation of cells [107]. It was reported that the culture of PC-3 cells treated with lutein extract decreased in proliferation, modulating the expression of growth genes associated with prostate cancer cells. Besides, results showed a synergic between lutein- and drug-induced effects with cell cycle arrest and apoptosis in prostate cancer [98]. Shree et al. [103] found that β-carotene induced apoptosis in MCF cells by caspase-3 activity and inhibited the expression of the anti-apoptotic proteins, Bcl-2 and PARP.

In addition, carotenoids have excellent quenching activity for singlet oxygen and lipid peroxidation. The mechanism for quenching of singlet oxygen is a physical reaction. Carotenoids take up thermal energy from singlet oxygen and release it by polyene vibration [107]. Levy et al. [104] demonstrated that supplementation with 60 mg/d of β-carotene to patients for 3 weeks resulted in a reduction in LDL susceptibility to oxidation, exhibiting a decrease in malondialdehyde (MDA) and lipid peroxides (PD) generation by 25 and 40%, respectively. As well, carotenoid intake may play a role in protecting telomeres by oxidative stress reduction. In a cross-sectional observational study, Boccardi et al. [105] associated the presence of β-carotene in plasma with telomerase activity in Alzheimer disease (AD) patients, since subjects affected by AD had significantly lower plasmatic levels of β-carotene (448 ± 66 mg/ml), as compared with healthy controls. 

In animal models, the effect of lutein as a neuroprotector and modulator of oxidative stress has been reported. Qiu et al. [102] reported that rats supplemented with lutein (0, 12.5, 25, or 50 mg/kg) for 45 days significantly improved body weight, total cholesterol and triglycerides accumulation, and insulin sensitivity. Similarly, Binawade et al. [101] demonstrated that rats supplemented with lutein (50–100 mg/kg) for 14 days improved fat loss and improved hind-limb impairment, motor coordination, and memory alterations. Also, the levels of lipid peroxidation, nitrite concentration, and glutathione in the rat brain were reduced.

Table 4 summarizes the main health benefits of carotenoids found in kale. Human studies have found no significant side effects associated with the intake of 20 mg/d of β-carotene and lutein [108,109,110,111].

## 3. Application of Controlled Abiotic Stresses as a Tool to Induce an Increase in the Content of Bioactive Compounds in Kale

Traditionally, genetic engineering has been applied to increase the expression level of genes and consequently the production of metabolites of interest in plants. However, this technology is complex and has been proposed as a potential biological hazard, limiting its commercial use for a few crops [112]. The application of abiotic stresses (i.e., wounding, modified atmospheres, temperature, soil composition, and phytohormones application) in fruits and vegetables has received great attention because they allow the accumulation of bioactive compounds with health-promoting properties [9,113].

Plant stress has been defined as a state where the plant is growing in non-ideal conditions, which induce an adaptive process and plant responses to these external factors or stressors that, in turn, cause a mixture of eustress and distress in the plant [114]. The eustress is a beneficial and reversible plant stress induced by a low or moderate exposition to a stressor. This process can modulate plant metabolism inducing the synthesis and enhancing the accumulation of beneficial secondary metabolites, improving the plant defense system [115]. The application of stress or eustress needs to be a controlled process to avoid over-activation of the defense system that could have an adverse effect on plant growth [116,117].

Since plants are sessile organisms, they need constant monitoring of environmental changes to modify and adjust parameters associated with development and metabolism. The response to these environmental stimuli requires an integrated mechanism, where internal and external signals are detected and cause an appropriate reaction in the plant [118]. The perception of stress involves the amplification of a stimulus by the transduction machinery composed of protein kinases, phosphatases, and binding proteins. Once amplified in the cytoplasm, the stress signal is transduced to the nucleus, where it would stimulate the expression of genes implicated in the primary and secondary metabolism of the plant, as well as a late reaction related to an increase in the enzyme activity implied in the biosynthesis and accumulation of secondary metabolites [10,112].

The responses of plants to abiotic stresses can be divided into an immediate and a late response. The immediate response is associated with the production of stress signaling molecules [i.e., reactive oxygen species (ROS), ethylene, jasmonic acid, methyl jasmonate (MeJA), etc.] that activate the expression of genes involved in the primary and secondary metabolism of the plant. On the other hand, the late response is associated with an increase in enzymes involved in the biosynthesis of secondary metabolites and the accumulation of secondary metabolites [10,110]. In the following sections, strategies to enhance the phytochemical composition of kale using pre-harvest and post-harvest controlled abiotic stresses, as described (Table 5).

### 3.1. Saline Stress Conditions

#### 3.1.1. Sulfur as an Abiotic Stressor

Sulfur (S) is a key element that plays a pivotal role in plant growth and development. The management of sulfur in crop plant nutrition is essential due to its crucial role in fundamental processes, such as homeostasis, electron transport, catalysis, and regulation [111].

Similarly to other macronutrients, S is taken up by the plant through the root as sulfate (SO_4_^2−^). To be incorporated in the metabolic pathways, sulfate is first activated by ATP sulfurylase to yield adenosine-5′-phosphosulfate (APS), which is then reduced to sulfite (SO_3_^2−^) by APS reductase. Finally, sulfite reductase converts the sulfite into sulfide that reacts with O-acetylserine in the presence of O-acetylserine lyase (OAS-TL) to produce Cys. From Cys, GSH is produced by two-step ATP-dependent reactions, where Cys is converted to γ-glutamylcysteine by γ-glutamylcysteine synthetase (also known as glutamate-cysteine ligase, GCL), and the subsequent reaction is catalyzed by glutathione synthetase. Cysteine also serves as a precursor of methionine (Met). Homocysteine is produced from cysteine and *O*-phosphohomoserine by the action of cystathionine γ-synthase (CGS) and cysta-thionine β-lyase (CBL). Homocysteine is then converted into Met by methionine synthase (MS). Methionine is the primary precursor of glucosinolate synthesis pathway by initiating the side chain elongation reaction to Met [119].

Glucosinolates are sulfur-rich anionic secondary metabolites, and therefore their concentrations in vegetables are influenced by the addition of S fertilizer [120]. The influence of S on glucosinolates content in the *Brassicaceae* family has been widely studied. Park et al. [121] performed a study to evaluate if sulfur positively affects the glucosinolate concentration in kale to enhance its health-promoting properties. The research required the germination of the kale seeds, ‘TBC.’ From 40 days after sowing, leaves were supplemented every 2 days with a sulfur (S) solution (0.0, 0.5, 1.0- and 2.0-mM) for 28 days. Individual and total glucosinolate content increased directly proportional to the S concentration. The maximum levels of total GLSs (26.8 mmol/g DW) and glucobrassicin (9.98 mmol/g DW) were found in the leaves supplemented with 2 mM S. Aliphatic glucosinolates, and total glucosinolates increased by 67% and 35%, respectively. Glucobrassicin was the main glucosinolate accumulated. Therefore, Park et al. [121] concluded that sulphur fertilizers constitute a method to enhance the anticancer phytochemical yields in young plants when soil nutrients are limiting.

Similarly, Kopsell et al. [122] evaluated the effect of sulfur supplementation of three kale cultivars, Winterbor, Redbor, and Toscano, to consider the variability previously reported for glucosinolates and carotenoid accumulation. During the study, the 2-week-old plants were treated with a solution supplemented with 4, 8, 16, 32, and 64 mg of S/L for 45 days. The contents of glucoiberin, glucobrassicin, neoglucobrassicin, and 4-hydroxygluco-brassicin increased significantly in all three cultivars, mainly in the leaves supplemented with 16, 32, and 64 mg of S/L. Glucobrassicin was the most abundant glucosinolate, showing an increase of 505% (274.3 mg/g DW), 746% (335.2 mg/g DW), and 362% (261.5 mg/g DW) in the Winterbor, Redbor, and Toscano cultivar, respectively, when compared with the 4 mg S/L treatment. However, there was no significant change in carotenoid accumulation by S treatment. Kopsell et al. [122] suggest that glucosinolate accumulation appears to be determined by S availability, cultivar, and their growing individual characteristics.

#### 3.1.2. Selenium as an Abiotic Stressor

Selenium (Se) is a trace mineral element essential in human and animal nutrition because it acts as a cofactor of selenoenzymes, such as glutathione peroxidase (GPx) and thioredoxin reductase (TrxR). These enzymes reduce reactive oxygen species (ROS) levels and maintain the redox balance in the human body [139]. Selenium has been classified as a chemopreventive agent due to its ability to reduce the risk, delay the progression, and avoid the cancer recurrence [140,141]. Despite its importance, Se deficiency in food is a common nutritional problem in various parts of the world [142].

Germination in the presence of Se condition could trigger sulfur (S) metabolic pathways due to their chemical similarity. Selenium forms (elemental, selenide, selenate, selenite, organic) present in nature determine its solubility and bioavailability in plants. Both selenate and selenite are the chemical forms predominantly absorbed by plants; however, selenate is the most mobile form within the plant. Selenate is probably absorbed in the root by sulfate transporters, located on the plasma membrane of the cell via S channels (SULTR1;2, SULTR1;1, and SULTR3;1). Apparently, it can enter leaf mesophyll cells by SULTR1;1 or SULTR1;2 and enters chloroplasts via SULTR3;1. Due to the similarity with S, selenate could enter the S metabolic pathways where would be converted in Se-amino acids. Once the inorganic Se enters the plastid, it must be converted by ATP sulfurylase (ATPS) in phosphoselenate (APSe). Then, the APSe is further reduced to selenite by the activity of APS reductase (APR). The conversion of selenite to selenide (Se^−2^) is reduced by sulfite reductase (SiR) or by the interaction with reduced adenosine phosphosulfate (GSH). In the last case, selenite and GSH are converted nonenzymatically to selenodiglutatione (GSSeSG), which is then transformed to selenopersulfide (GSSeH) and finally to selenide through glutathione reductase (GR). Thereafter, Se^−2^ is incorporated into SeCys via the cysteine synthase (CS) complex, which consists of the enzyme serine acetyltransferase (SAT), its product, *O*-acetylserine (OAS), and the *O*-acetylserine thiol lyase (OASTL) enzyme [143].

**Figure 3:**
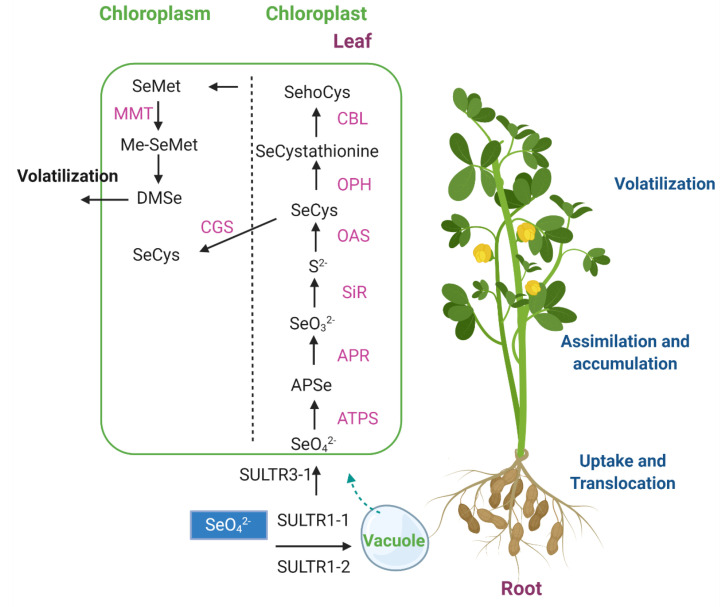
Scheme of main steps in selenium (Se) metabolism. SULTR: sulfate transporter; ATPS: ATP sulfurylase; APR: APS reductase; SiR: sulfite reductase; OAS: *O*-Acetyl Serine. OPH: *O*-phosphohomoserine; CBL: cystathione beta-lyase; MMT: S-adenosyl-L-Met:Met-S-methyltransferase; CGS: cystathione-c-synthase. Figure created with BioRender.com (accessed on 20 October 2021).

Likewise, the amino acid SeCys can be transformed to SeMet in three enzymatic steps. Briefly, SeCys is converted to selenocystathione (Se-cystathionine) through *O*-phosphohomoserine (OPH) and SeCys, which is catalyzed by cystathione-c-synthase (CGS) [143]. Then, Se-cystathionine may be converted to selenohomocysteine (Se-homocysteine) by cystathione beta-lyase (CBL) [143]. Finally, methionine synthase (Met synthase) uses methyl-tetrahydrofolate as a carbon donor to convert Se-homocysteine into SeMet. Once more, the plant can volatize Se from SeMet by methylation to form methyl-selenometionina (SeMet) via S-adenosyl-L-Met:Met-S-methyltransferase (MMT), and then the conversion to DMSe by methylmethionine hydrolase [143]. A schematic representation of selenium metabolism in plants is shown in Figure 3. Se also can be incorporated into (seleno)glutathione, glucosinolates, and iron (Fe)-Se clusters. Se supplementation has been shown to up-regulate the secondary metabolism of plants that involves enzymatic and non-enzymatic antioxidants [144].

In adequate concentrations, the role of Se consists of soil nutrient enrichment, regulation of ROS, translocation of heavy metal, and restoring the cell membrane and chloroplast structures in plants. However, an excess of Se triggers the accumulation of ROS [145].

The production of ROS at high Se levels may be partially related to an imbalance in the levels of GSH, thiols (-SH), and NADPH, which can play a vital role in the oxidizing cell environment. All these signaling molecules lead to the overexpression of stress-related genes, normally induced by defense signaling pathways (e.g., *PR-1*, *PR-2*, *PR-5*, and the defense gene *PDF1.2*) [146]. The high level of ROS upregulates the expression of several genes involved in phenolic biosynthesis (e.g., the maize transcription factor *ZmP* and *MYB12,* and their target genes *CHS* and *CHI* –chalcone isomerase).

Plants of the *Brassicaceae* family can store Se at concentrations of up to 10–15 mg Se/g DW in their shoots while growing on soils containing only 0.2–10 mg Se/kg. The non-specific integration of Se into the S assimilation pathway enables the plant to metabolize selenoamino acids, selenocysteine, and selenomethionine into proteins [147].

There are few reports of the effect of selenium supplementation on the concentration of bioactive compounds present in kale plants. Kim et al. [123] evaluated the effects of sodium selenite on glucosinolates and ITC content in kale (*Brassica oleracea* var. *sabellica*). Six-week-old kale plants were exposed to 2 mg/L Na_2_SeO_3_, 80 mM NaCl, or a combination for 2 weeks. The results showed that kale roots accumulated higher levels of gluconasturtiin with NaCl (15%, 9.1 µmol/g DW), Na_2_SeO_3_ (19%, 11.8 µmol/g DW), or both (27%, 16.4% µmol/g DW) after seven days of treatment; however, there was no statistically significant difference in the glucoraphanin content between the control and treated kale plant. In addition, the ITC concentration was increased 30% (6 µmol/g DW) by a 2-week treatment with the combination of both compounds. Kim et al. [123] suggest that kale is a plant with a wide range of salt tolerance, and the elicitation of their individual glucosinolates responds differentially to it.

#### 3.1.3. NaCl as an Abiotic Stressor

Salinity is one of the most severe stress factors that limit crop production. It can cause two kinds of stress on plant tissues: ionic and osmotic [148]. Ionic stress is associated with high Na^+^/K^+^ and Na^+^/Ca^+2^ ratios and the accumulation of Na^+^ and Cl^-^ in tissues, which is harmful to the general metabolism of cells [149]. Osmotic stress occurs when the increase in salts in the soil solution causes a decrease in the hydric and osmotic potentials of the soil, which is reflected in the hydric state of the plant or relative water content. The plant tends to lose water; thus, the plant must maintain a more negative water potential than the substrate to ensure water absorption [148].

Increasing osmotic stress in plants produces stomatal closure to prevent water loss, causing a reduction of CO_2_ [150]. The direct response of the Calvin cycle to such situations leads to oxidized NADP^+^. The deficiency of NADP^+^, which serves as an electron acceptor in photosynthesis, is the underlying cause of electron donation from over-reduced ferredoxin to oxygen, forming superoxide radicals by the Mehler reaction [151]. The formation of ROS in salt-stressed plants triggers defense mechanisms, resulting in the accumulation of a wide range of enzymatic and non-enzymatic antioxidants which can quench the ROS [152].

Differences in the glucosinolate metabolism of kale may occur under salinity stress. Wang et al. [124] evaluated the effect of NaCl in kale sprouts (*Brassica oleracea* var. *alboglabra*) on glucosinolate, ascorbic acid, and carotenoid content. Five-day-old kale sprouts were treated with a 160 mM NaCl solution for two days. There was a significant increment in the total glucosinolate (129%) accumulation by NaCl treatment, especially aliphatic glucosinolates (142%). The levels of glucoiberin, glucoraphanin, glucoerucin, gluconapin, and progoitrin were increased by 190% (0.29 µmol/g FW), 144% (1.32 µmol/g FW), 166% (0.40 µmol/g FW), 175% (4.71 µmol/g FW), and 10% (0.55 µmol/g DW), respectively, when compared with the control. In addition, the indole glucosinolate glucobrassicin increased by 233% (0.10 µmol/g FW). The content of ascorbic acid and total carotenoids was 83% (62.5 mg/100 g FW) and 53% (3.5 mg/100 g FW), respectively, and higher in treated sprouts. However, the total phenolics and antioxidant capacity were not affected by the NaCl treatment.

The individual phenolic response has also been associated with a response to saline stress. Linic et al. [125] evaluated the content of chlorophyll and individual phenolics in kale (*Brassica oleracea* var. *acephala*) grown in agar plates containing NaCl (50–200 mM). Levels of salicylic acid (27.5%, 59.21 pmol/mg DW), caffeic acid (31.1%, 114.07 pmol/mg DW), and 4-coumaric acid (108.1%, 90.85 pmol/mg DW) decreased, while ferulic acid (19%, 302.53 pmol/mg DW) increased in kale under severe stress (200 mM NaCl). However, the treatment did not affect the concentration of chlorophyll pigments in kale.

### 3.2. Methyl Jasmonate as an Abiotic Stressor

**Figure 4:**
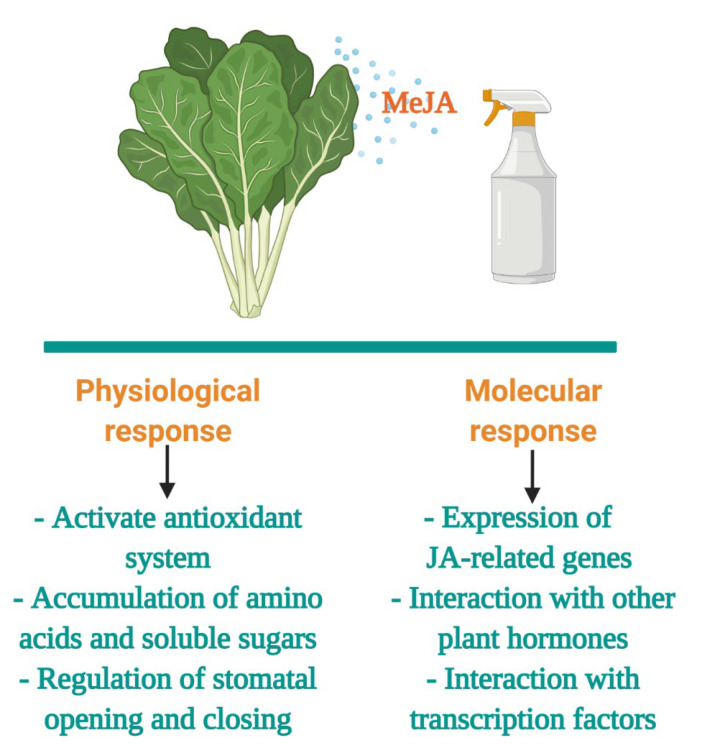
General mechanisms and functions in methyl jasmonate (MeJA) stress tolerance of plants. Figure created with BioRender.com (accessed on 20 October 2021).

Phytohormones, such as MeJA, are another class of abiotic stressors that trigger cascades of physiological and molecular responses, resulting in the synthesis and accumulation of secondary metabolites [153]. These responses often involve activation of the antioxidant system (superoxide anion radical, peroxidase, and NADPH-oxidase), accumulation of amino acids (isoleucine and methionine), and soluble sugars, and regulation of stomatal opening and closing. In addition, the expression of genes implicated in secondary metabolism, cell-wall formation, and defense-related are upregulated [154]. Thus, exogenous phytohormones can be applied to plants as an approach to enhance their phytochemical content. A summary of the mechanisms of MeJA in abiotic stress tolerance is shown in Figure 4. 

The effects of MeJA on the phytochemical profile in plants have been focused on the accumulation of glucosinolates. For instance, it is known that there are several transcription factors identified, which regulate the biosynthesis of glucosinolates in Arabidopsis and other related Brassica species, including *OBP2*, also called *AtDof1.1* (DNA- binding-with-one-finger). *OBP2* is a positive regulator of the network controlling indole glucosinolate biosynthesis in Arabidopsis [155]. In Arabidopsis plants, the expression of OBP2 is stimulated by wounding, MeJA treatment, and in response to insect feeding, leading to an induced expression of glucosinolate biosynthetic genes and a subsequent accumulation of glucosinolates. In addition, the overexpression of OBP2 in transgenic plants resulted in the upregulation of *CYP79B2/B3* and *CYP83B1* genes involved in the glucosinolate biosynthetic pathway and an increase in indole glucosinolates [156]. Moreover, it has been reported that MeJA treatment induces the accumulation of all types of indolyl glucosinolates, as it can induce other transcription factors involved in the indole glucosinolate pathway (i.e., *MYB51* and *MYB34*), subsequently altering the expression levels of *CYP79B2/B3* and *SOT16*, and finally resulting in enhanced indolyl glucosinolate levels [157]. Regarding other types of glucosinolates, MeJA can only induce the aliphatic transcription factor *MYB28*, while it has no response to other aliphatic regulators, such as *MYB29* and *MYB76* [158].

Sun et al. [126] performed a study to evaluate whether MeJA induces an effect in the content of glucosinolates in kale. MeJA (100 μM) was applied by vapor fumigation in thirty-day-old kale plants. The MeJA treatment induced a significant increase in the concentrations of glucobrassicin (520%, 4.25 μmol/g DW), neoglucobrassicin (1420%, 3.16 μmol/g DW), and total indole glucosinolates (230%, 13.2 μmol/g DW), when compared with control at 1 d after treatment.

Yi et al. [128] obtained similar results in four-month-old leaf kale sprayed with 250 μM MeJA. The application of the MeJA treatment induced an increase of indolyl and aliphatic glucosinolates in kale, including glucoraphanin (735%, 1.67 μmol/g DW) glucobrassicin (1708%, 4.52 μmol/g DW), and neoglucobrassicin (1800%, 0.38 μmol/g DW). In addition, gene expression levels of *ST5a (Bol026200), CYP81F1* (*Bol028913, Bol028914*), and *CYP81F4* were significantly upregulated, which suggests that the metabolic changes promoted by MeJA application share common activation mechanisms with the insect herbivory response [128,159].

Few studies have been conducted regarding the elicitor effects of MeJA on the accumulation of phenolic compounds and carotenoids in kale and other plant species. For instance, Ku and Juvik [127] observed that the exogenous application of MeJA (250 μM) in two kale cultivars, Red Winter (*Brassica napus* var. *pabularia)* and Dwarf Blue Curled Vates (*Brassica oleracea* var. *acephala*), significantly increased total phenolics by 27% at commercial maturity.

### 3.3. Drought as an Abiotic Stressor

Drought stress, defined as a naturally occurring water deficit, is one of the leading causes of crop losses in the agricultural world. As with other forms of stress, the plant creates drought adaptation mechanisms resulting in an excessive ROS accumulation. The strategies of plants to deal with drought stress are divided into three mechanisms: the ability of plants to complete their life cycle before the water deficit becomes more severe, by selecting for earliness; the ability of plants to maintain a relatively high water potential under conditions of water stress, through stomatal closure and osmotic adjustment; and the ability of plants to reduce the chemical activity of water, through the concentration of solutes and macromolecules.

Chloroplasts have received particular attention in the literature on oxidative stress due to their importance for generating ROS [160,161]. The general effects of drought on the photosynthetic machinery provide two important sources of ROS: first, it is expected that any reduction in normal photosynthetic function (stomatal closure and CO_2_ availability) favors the generation of singlet oxygen in the photosystem II (PSII) [162,163]. On the other hand, the malfunction of the electronic transport systems should promote a net flow of electrons towards the molecular oxygen (Mehler reaction), thus favoring the synthesis of superoxide and H_2_O_2_ [151].

Barickman et al. [129] evaluated three different levels of volumetric water content (VWC) (0.15, 0.25, or 0.35 m^3^) in Kale ‘Winterbor’ plants. Results showed that kale grown in conditions of medium and high water deficiency (0.25 and 0.35 m^3^, respectively) presented a decrease in the carotenoids neoxanthin (16.6%, 0.193 μg/g DW) and antheraxanthin (18.2%, 0.033 μg/g DW). Additionally, the glucosinolates glucoiberin (53%, 8.74 μmol/g DW), progoitrin (60%, 0.08 μmol/g DW), and total phenolic content (7.89 mg/g DW) were the highest concentration in the medium water deficiency level. Similarly, Yoon et al. [130] evaluated drought stress in five-week-old kale (*Brassica oleracea* var. *acephala*) by removing all the nutrient solution from the root system 7 days before harvest. Their results showed that less than 4 days could ensure the function of leaf chlorophyll fluorescence and maintain normal leaf water potential. In addition, total flavonoid content (3.2 mg/g), total phenolic (8.1 mg/g) content, and antioxidant activities (3.9 mg/g) increased significantly by 35%, 48%, and 34%, respectively, on days 3 and 4 before harvest. Both studies suggest that medium drought stress conditions can enhance plant tolerance and improve the antioxidant system.

Furthermore, the effect of drought stress on the physiological parameters of kale has been previously reported. Issarakraisila et al. [164] investigated the physiological and growth responses of kale (*Brassica oleracea* var. *alboglabra*) to water deficit for 19 days. The water deficit reduced leaf area (86%), leaf number (38%), fresh weight (90%), and dry weight (80%). The water deficit increased the nitrogen concentration in the leaf dry matter by more than 60% and produced closed stomata.

Although the application of water stress as an approach to enhance the phytochemical content has been reported in kale, the research mainly focuses on physiological and biochemical parameters rather than nutraceutical-related applications [164,165].

### 3.4. Temperature as an Abiotic Stressor

Most plant species are sensitive to temperature and are under stress when it is low or high with respect to the thresholds defined for each one. In general, four types of heat stress are recognized in plants: sustained high temperatures, frequent episodes of high temperatures (“heat shock”), chilling injury (0 to 10 °C), and damage by freezing at temperatures below 0 °C, which causes the formation of ice in plant tissues [166].

High temperatures generate anatomical, morphological, and functional changes in plants, similar to those produced by water stress, including: reduction in cell size, reduced stomatal conductance and stomatal closure, changes in membrane permeability, increases in stomatal and trichome density, and larger xylem vessels [167]. 

Cell membranes are the first to be affected by heat stress [168]. The increase in temperature leads to denaturation of proteins and enzymes and increases in the amount of unsaturated fatty acids [169], causing the lipids of the membranes to become more fluid and permeable and allowing the loss of electrolytes [170,171]. 

The symptoms of heat damage may initially manifest as deviations in carbohydrate content, alterations in the levels of growth regulators, accumulation of toxic substances, color changes, and the development of malformations. In addition to accelerating cellular metabolism and causing tissue dehydration, heat stress generates oxidative stress because it induces the production of reactive oxygen species (ROS) [166]. Plants respond with the production of antioxidant enzymes, such as superoxide dismutase, ascorbate peroxidase, glutathione reductase, and catalases [172,173], and cascades of signals that induce the expression of defense genes are triggered [167].

On the other hand, the effects of cold stress on plants produce the called “phase transition”. First, the structural changes associated with low-temperature stress involve a decrease in the fluidity of the membranes. This breaks up the homeostasis of the cell by impeding the ionic gradient [174]. Secondly, structural changes associated with low-temperature stress, such as the deformation of the thylakoids, swelling of the chloroplasts, and mitochondria, reduce the efficiency of photosynthesis [175]. Thirdly, due to increased anaerobic respiration, signaling molecules, such as ethylene and ROS, accumulate in plants at low temperatures [176,177]. 

Despite the negative effect of a low temperature on plants, it can be used to induce resistance mechanisms and overproduce secondary metabolites [158]. For example, the activation of antioxidant enzymes (SOD, CAT, APX, and GR) and elicitation of secondary metabolites (e.g., phenolic compounds and carotenoids) protects plant cells from oxidative damage by scavenging ROS [131].

Recently, short-term temperature stress to crops during cultivation has been considered a strategy to increase the levels of health-promoting phytochemicals in plants. Manchoo Collard kale exposed to 4 °C for 3 days exhibited a 15% higher total phenolic concentration (~0.5 mg GAE/g FW) and 17% higher antioxidant capacity (~1.5 mg TEAC/g FW) than the control after treatment, whereas that of ‘TBC’ were both 16% lower than control. Individual phenolic compounds, such as caffeic acid, ferulic acid, and kaempferol, exhibited a similar trend to the total phenolic concentration [131]. Jurkow et al. [132] evaluated cold weather conditions (>0 °C, −5.0 °C, and −15.0 °C) in Winterbor and Redbor. The results showed that the content of ascorbic acid (27% and 14%), phenolics (60% and 90%), and antioxidant activity (340% and 80%) reached their maximum point after −15.0 °C frost in Winterbor and Redbor cultivars, respectively. Likewise, the level of anthocyanins also increased significantly for Redbor cultivar by 511% (110 mg cy-3-glu/100 g FW) and 400% (110 mg cy-3-glu/100 g FW) with medium and heavy frost, respectively. Carotenoids significantly increased in both Winterbor and Redbor with medium treatment, 55% (0.221 mg/g FW) and 22.3% (0.296 mg/g FW), respectively.

Heat-shock treatment has also been reported to increase the amount of antioxidant and anti-carcinogenic compounds in kale. Lee et al. [134] evaluated the effect of heat-shocks (50 °C) in water for 10, 20, 30, 45, or 60 s in four-day-old kale sprouts. Kale treated by 50 °C/20 s heat-shock showed a 150% higher total phenolic (0.99 mg GAE/g FW) concentration and a 120% higher antioxidant capacity (4.3 mM TEAC/g FW) than the control. In addition, the accumulation of total glucosinolates significantly increased (33%, 120 mmol/g DW) with the same treatment.

### 3.5. Radiation as an Abiotic Stressor

Solar radiation is essential for life on earth. Higher plants use sunlight to direct and regulate fundamental processes, such as germination, growth and development, photosynthesis, and flowering [178]. The components of the electromagnetic spectrum that participate in these processes include visible light, ultraviolet (UV), and infrared radiation. Based on the wavelengths it encompasses, UV radiation is divided into three regions: UV-A (320–400 nm) is the least harmful range; UV-B (280–320 nm) causes several detrimental effects in plants; and UV-C (100–280 nm) is completely absorbed by stratospheric ozone [179].

Two signaling pathways regarding how plants perceive UV-B radiation and regulate secondary plant metabolism have been proposed. One pathway is non-specific to UV-B and implies the accumulation of signaling molecules, such as ROS, jasmonic acid (JA), salicylic acid (SA), and nitric oxide (NO). They all lead to over-expression of stress related genes, normally induced by defense signaling pathways (e.g., *PR-1*, *PR-2*, *PR-5* and the defense gene *PDF1.2*) [146,179].

In contrast, the signaling pathways that mediate responses to UV-B as a signal appear to be UV-B-specific and result in UV-protection or morphological changes [180]. In the presence of UV-B, cytosolic UVR8 photoreceptor monomerizes and interacts with the multifunctional E3 ubiquitin ligase constitutively photomorphogenic 1 (COP1) and translocate into the nucleus, where they prevent the degradation of the photomorphogenic transcription factor elongated hypocotyl 5 (HY5). Successively, *HY5* and its homolog (*HYH*) control expression of a range of key elements involved in UV acclimation response and UV protection, such as gene-encoding enzymes of the phenylpropanoid pathway, e.g., phenylalanine ammonia-lyase (PAL), chalcone synthase (CHS), and flavonol synthase (FLS) [146,181,182,183,184,185].

Despite the evident relevance of UV-A radiation on plant morphology, physiology, biochemistry, and photosynthesis, there is a lack of scientific studies to elucidate the signaling mechanisms governing such responses [186]. Although the application of UV stress as an approach to enhance the phytochemical content has been reported in *Brassica* plants, including several reports on broccoli [138,187,188], less is known about the effect of UV on kale.

In kale plants, the accumulation of glucosinolates, total phenolics, and flavonoids has is stimulated by exposure to radiation. Alegra et al. [135] reported a significant increase in aliphatic glucosinolates in Half Tall and Black Magic kale radiated after two days of germination with high light (800 μmol photons/m^2^ s). Black Magic showed an increase in glucoraphanin (150%, 100 nmol/g FW), glucoerucin (350%, 1.8 nmol/g FW), and total aliphatic glucosinolates (175%, 110 nmol/g FW) by high light treatment, while Half Tall displayed the same response to treatment in glucoberverin (2%, 6 nmol/g FW), glucoraphanin (400%, 20 nmol/g FW), glucoerucin (100%, 0.5 nmol/g FW), and total aliphatic glucosinolates (66%, 100 nmol/g FW).

Studies of the effect of UV-A and UV-B radiation on kale report an increase in phenolics and flavonoids. Klopsch [137] evaluated the effect of radiation UV-B (0.0189 W/m^2^) and UV-A (69.502 W/m^2^) for 2 h daily in ten-day-old kale sprouts. The results showed a deviation of metabolic resources towards the biosynthesis of quercetin-3,7,4 ′-*O*-d-triglucoside (0.624 mg/g FW), quercetin-3-*O*-caffeoyl-sophoroside-7-*O*-glucoside (0.195 mg/g FW), and quercetin-3-*O*-sinapoyl-sophoroside-7-*O*-d-glucoside (0.175 mg/g FW), which significantly increased by 26%, 30% and 33%, respectively, compared to the control. The antioxidant activity increased proportionally.

Likewise, Lee et al. [131] evaluated a 5-week-old cultivar under two UV-A radiation treatments (370 and 385 nm) for 5 days to test changes in the total and individual phenolics. The authors found that total phenolics (1.0 and 0.9 mg GAE/g FW) showed a significant increase (25% and 42%) in response to 370 nm and 385 nm, respectively, at 5 days of treatment. The levels of caffeic acid (200% and 180%) and kaempferol (146% and 168%) were significantly increased by 370 and 385 nm UV-A treatments, respectively.

## 4. Drawbacks of Using Abiotic Stresses at a Large Scale to Increase the Content of Bioactive Compounds in Kale

The application of abiotic stressors at larger scales remains labor intensive, especially in larger field areas and dense crop plantations. In addition, the dose-optimization requires variety-specific studies [189]. Further, under field conditions, various stresses occur in combinations that magnify stress severity. According to the National Climate Assessment—USDA, abiotic stresses can generate high losses in global crop production (~50%) [190].

In addition, it is necessary to consider the possible accumulation of anti-nutritional factors present in kale due to exposure to abiotic stress. Kale has been reported as a source of oxalates, nitrates, tannin, and phytate. These compounds have a strong binding affinity to minerals, such as calcium, magnesium, iron, copper, and zinc, making them unavailable for absorption in the intestines [191]. Quality attributes of the crop, which are relevant for consumers, could also be affected by the application of pre- and post-harvest abiotic stresses [192]. Thus, the use of non-thermal technologies has been recently proposed as an effective tool to increase the content of health-promoting compounds in vegetables, while retaining quality attributes [192].

## 5. Conclusions

In this review, the health benefits of kale related to its main phytochemicals (phenolics, carotenoids, ascorbic acid, and glucosinolates) were discussed based on recent in vitro, in vivo, and clinical studies. Literature supports that kale can be considered a super-food due to its high content of phytochemicals and several studies supporting their pharmacological activity. Herein, different controlled abiotic stress conditions that affect the content of secondary metabolites of nutraceutical importance in kale were also discussed. For instance, saline stress (S, Na2SeO3, and NaCl) and exogenous phytohormone (MeJA) can be applied to improve glucosinolate concentration. Moreover, it was observed that the phenolic content could be improved with UV-A and UV-B radiation or exposure to short-term temperature stress. Finally, carotenoid content (mainly xanthophylls) can be positively affected by medium drought stress, whereas they can be degraded by cold and heat stress. Despite all the evidence validating secondary metabolism elicitation through abiotic stresses, it is not yet applied at an industrial scale to produce kale. Thus, it is highly relevant to transfer this knowledge to kale producers to generate a product with a higher potential to prevent chronic and degenerative diseases. The information reviewed in this article can be used as a starting point to validate the effects of abiotically stressed kale on the prevention and treatment of chronic and degenerative diseases through bioassays.

## Figures and Tables

**Table 1:** Predominant glucosinolates in kale and their related isothiocyanates (ITCs).

Glucosinolates		
Side Chain Name	Trivial Name	Isothiocyanate
Methylthiobutyl	Glucoerucin	Erucin
3-Methylsulfinylpropyl	Glucoiberin	Iberin
3-Methylsulfinylbutyl	Glucoraphanin	Sulforaphane
2-Phenylethyl	Gluconasturtiin	Phenylethyl ITC
3-Indolylmethyl	Glucobrassicin	indol-3-carbinol
2-Hydroxyl-3-butenyl	Progoitrin	2-hydroxyalkenyl

**Table 2:** Health benefits of isothiocyanates (ITC) found in kale, determined through in vivo and in vitro studies.

Compound	Metabolic Effect	Main Findings	Reference
Sulforaphane (SFN)	Nrf2 Mitigate insulin resistance	C57BL/6JSlc mice were supplemented with 0.3% glucoraphanin for 14 weeks. Results showed diminished weight gain, reduced hepatic steatosis, and improved insulin sensitivity.	[47]
	C/EBPα Adipocyte differentiation	3T3-L1 pre-adipocytes cell line treated with SFN inhibited the elevation of triglycerides in the adipocytes by activation of PPARγ and (C/EBP) α, and inhibition of (C/EBP) β. SFN arrested the cell cycle at the G0/G1 phase.	[48]
	C/EBPα	3T3-L1 pre-adipocytes cell line treated with SFN (10 μM) showed significant inhibition of adipocyte differentiation and lipid accumulation by degradation of CCAAT/enhancer-binding protein (C/EBP) β.	[50]
Benzyl isothiocyanate Phenethyl isothiocyanate	Adipocyte differentiation	C57BL/6J mice were fed with 1g/kg of benzyl isothiocyanate or phenethyl isothiocyanate for 18 weeks. Results showed that these isothiocyanates have the potential to prevent body weight gain.	[54]
Allyl isothiocyanate	Sirt1/AMPK NF-κB	AML-12 mouse hepatocyte cell line was treated with allyl isothiocyanate (20 μmol/L) for 24 h. Allyl isothiocyanate reduced lipid accumulation and inflammation in vitro through the Sirt1/AMPK and NF-κB signaling pathways.	[55]
Phenethyl isothiocyanate	Hormone regulation	C57BL/6J mice were fed with 25 mg/kg phenethyl isothiocyanate for 18 weeks. Results showed that phenethyl isothiocyanate has the potential to stimulate hypothalamic leptin signaling.	[60]
Indol-3-carbinol (I3C)	Liver enzymes	C57BL/6J mice were fed with 40 mg/kg I3C for 35 days. Treatment modulated glucose tolerance and insulin sensibility. Also, supplementation with I3C increased SOD, CAT, GPx levels.	[61]
Indol-3-carbinol (I3C)	Apoptosis	I3C inhibited the phosphorylation and following activation of enzyme Akt kinase in PC-3 cell line. Akt kinase is involved in apoptosis and cell cycle regulation.	[62]
	Apoptosis	In vitro, I3C caused DNA strand breaks in three cervical cancer cell lines. (LD50) = 200 μmol/L I3C.	[63]
	Adipogenesis	Mice fed with a I3C-supplemented diet (1 g/kg diet) for 10 weeks showed significantly decreased expression levels of key adipogenic transcription factor PPARγ2, and its target genes, such as leptin and adipocyte protein 2.	[64]
	Anti-carcinogenic	MCF-7 cell line treated with I3C (10–125 μm/L) showed a significant inhibition of the ER-alpha signaling and the expression of the estrogen-responsive genes, *pS2*, and *cathepsin-D*. On the other hand, breast cancer susceptibility gene 1 (*BRCA1*) expression was upregulated.	[65]
	Anti-carcinogenic	Rats treated intraperitoneally with I3C showed significant inhibition in the development and metastases of prostate cancer and overall survival advantage.	[66]

Abbreviations: C/EBP) β: CCAAT enhancer binding protein beta; NF-κB: Nuclear factor kappa B; AML-12: alpha mouse liver 12 cell line; Sirt1/AMPK: histone/protein deacetylase/AMP-activated protein kinase; SOD: superoxide dismutase; CAT: catalase; GPx: glutathione peroxidase; I3C: Indol-3-carbinol; PC-3: Caucasian prostate adenocarcinoma; *BRCA1*: breast cancer 1; MCF-7: Michigan Cancer Foundation-*7 (*human breast cancer cell line); ER-alpha: Estrogen receptor alpha.

**Table 3:** Health benefits of the main phenolic compounds found in kale, determined through in vivo and in vitro studies.

Compound	Metabolic Effect	Main Findings	Reference
Quercetin	Antioxidant activity Neuroprotective	Cortical cells treated with quercetin (100 μM) inhibited up to 61% of the neurotoxicity produced by adding NMDA (n-methyl-d-aspartate) and kainate. In addition, quercetin showed a significant decrease in free radicals in brain injury caused by exposure to chemical agents.	[77]
	Vasodilator Normotensive, Normocholesteroleic	Healthy volunteers treated with quercetin (200–400 mg) showed an increase in brachial arterial diameter, demonstrating its vasodilator effects. That effect was correlated with an increase in plasma and urinary levels of glutathione.	[78]
Kaempferol and quercetin	Anti-inflammatory	The capacity of flavanoids wa proved to effectively inhibit the lipopolysaccharide (LPS)-induced PGE2 production, LPS-induced COX-2 expression, and mPGES-1 expression in activated macrophages.	[79]
	Antiproliferative	HepG2 cells exposed to a *Ginkgo biloba* leaf extract, kaempferol, and quercetin (50 and–100 μM) showed DNA damage and topoisomerase II inhibition.	[80]
Kaempferol- glucoside	Synergistic effect with *Lactobacillus paracasei* A221	The oral administration of kaempferol-rich kale extract to Sod1-deficient mice improved various pathologies, including skin thinning, fatty liver, and anemia.	[81]
Gallic, proto-catechuic,p-hydroxybenzoic, vanillic, salicylic,p-coumaric,caffeic, ferulic and sinapic acid	Antioxidant and anti-bacterial activities	Phenolic extracts of kale showed anti-bacterial effect on Gram-positive (*S. aureus, E. faecalis, B. subtilis*), Gram-negative (*M. catarrhalis*) bacteria, and two yeast-like fungi (*C. tropicali* and *C. albicans*).	[82]

**Table 4:** Health benefits of carotenoids found in kale, determined through in vivo and in vitro studies.

Compound	Metabolic Effect	Main Findings	Reference
Lutein	Antiproliferative	Lutein induced a decrease in the proliferation of PC-3 cells (in vitro), modulating the expression of growth genes associated with prostate cancer cells.	[98]
	Antioxidant activity	Healthy subjects supplemented with 12 mg/d of lutein for 12 weeks showed improved visual function, particularly in contrast sensitivity.	[99]
	Neuroprotective	Rats supplemented with lutein (30, 15, and 7.5 mg/kg) significantly elevated the activities of superoxide dismutase, glutathione peroxidase, and catalase in brain and decreased the neurological deficit scores.	[100]
	Neuroprotective	Rats supplemented with lutein (50–100 mg/kg) for 14 days showed a fat loss, reduced neurobehavioral alterations, and reduced oxidative stress.	[101]
	Antidiabetic and obesity control	Rats supplemented with lutein significantly improved body weight, hepatic levels of lipid accumulation, and insulin sensitivity.	[102]
β-carotene	Apoptotic	β-carotene (1 μM) induced apoptosis in MCF cells by caspase-3 activity and inhibited the expression of the anti-apoptotic proteins, Bcl-2 and PARP.	[103]
	Antioxidant activity	Patients supplemented with 60 mg/d of β-carotene for 3 weeks showed a reduction in LDL susceptibility to oxidation.	[104]
	Antioxidant activity	The content of β-carotene in plasma was significantly and positively correlated with telomerase activity of Alzheimer disease patients, independent of gender.	[105]

**Table 5:** Effect of different abiotic stress conditions on the accumulation of health-promoting compounds in kale.

Abiotic Stress	Treatment	Main Findings on the Biosynthesis of Phytochemicals	References
Saline stress	6-week-old kale seedlings were supplemented with sulphur (S) solution (0.0, 0.5, 1.0-, and 2.0-mM) for 28 days.	Maximum levels of total GLSs and glucobrassicin were found in the leaves supplemented with 2 mM S. Aliphatic glucosinolates, and total glucosinolates increased by 67% and 35%, respectively. Glucobrassicin was the main glucosinolate accumulated.	[121]
	2-week-old kale sprouts (Winterbor, Redbor, and Toscano) were treated with sulphur (S) solution (4, 8, 16, 32, and 64 mg/L) for 45 days.	There was a significant increase in the glucoiberin, glucobrassicin, neoglucobrassicin and 4-hydroxygluco-brassicin content in the leaves supplemented with 32 and 64 mg of S/L. Glucobrassicin was the most abundant glucosinolate. There was no significant change in carotenoid accumulation by S treatment.	[122]
	6-week-old kale seedlings (*Brassica oleracea* var. *sabellica*) were exposed to Na_2_SeO_3_ (2 mg/L), NaCl (80 mM), or a combination for 14 days.	After seven days of treatment, gluconasturtiin concentration increased by 15%, 19%, and 27% with NaCl, Na_2_SeO_3_, or both, respectively. ITC concentration increased 30% with the combination of both treatments after 14 days.	[123]
	Five-day-old kale sprouts (*Brassica oleracea* var. *alboglabra*) were treated with NaCl solution (160 mM) for two days.	Total glucosinolate increased (129%) by NaCl treatment, mainly aliphatic glucosinolates (142%). Glucobrassicin, glucoiberin, glucoraphanin, glucoerucin, gluconapin, and progoitrin increased by 233%, 190%, 144%, 166%, 175%, and 10%, respectively. Ascorbic acid and total carotenoids were 83% and 53% higher, respectively, in treated sprouts.	[124]
	Kale seedlings (*Brassica* *oleracea* var. *acephala*) were grown in 1% agar plates containing NaCl (50–200 mM)	There was no change in chlorophyll pigments in kale in the salt conditions. There was a decrease in salicylic acid (27.5%), caffeic acid (31.1%), and 4-coumaric acid (108.1%) in kale under severe stress (200 mM NaCl).	[125]
Phytohormone Application	Thirty-day-old chinese kale seeds (*Brassica* *oleracea* var. *alboglabra*) were sprayed with a MeJA solution (100 μM) 6 days before sampling.	Glucobrassicin (520%), neoglucobrassicin (1420%), and total indole glucosinolates (230%) showed a significant increase after 1 d of treatment. MeJA treatment did not exert a significant effect on the content of vitamin C, carotenoids or phenolics.	[126]
	Kale cultivars Red Winter and Dwarf Blue Curled Vates were sprayed with a MeJA solution (250 μM) 4 days before harvest at commercial maturity.	MeJA treatments significantly increased total phenolics in Dwarf Blue Curled (2298 mg GAE/100 g) and Red Winter (2070 mg GAE/100 g) cultivar by 24% and 41%, respectively. In addition, antioxidant activity also increased by 31% in both kale cultivars.	[127]
Phytohormone Application	Four-month-old kale plants were treated with a MeJA solution (250 μM) 4 days before sample collection.	Glucoraphanin (735%), glucobrassicin (1708%), and neoglucobrassicin (1800%) increase significantly. Higher expression of *ST5a* (Bol026200), *CYP81F1* (Bol028913, Bol028914), and *CYP81F4* genes were associated with this accumulation.	[128]
Drought	Kale ‘Winterbor’ were greenhouse growth at three different levels of volumetric water content (VWC): 0.15 m^3^, 0.25 m^3^, 0.35 m^3^.	Neoxanthin (16.6%) and antheraxanthin (18.2%) increased mainly with 0.35 m^3^ VWC treatment. Total phenolic content was higher with 0.25 and 0.35 m^3^ VWC treatments. Likewise, a higher concentration for glucoiberin (53%) and progoitrin (60%) was observed at 0.25 m^3^.	[129]
	Five-week-old kale plants (*Brassica oleracea* var*. acephala*) were stressed by removing the nutrient solutions 7 days before harvest.	Total phenolic and flavonoid contents and antioxidant activities were significantly increased by 35%, 48%, and 34%, respectively, in treated samples at 3–4 days.	[130]
Temperature	Five-week-old kale (Manchoo Collard) were subjected to 4 °C for 3 days.	Treated kale ‘Manchoo Collard’ exhibited a 15% higher total phenolic concentration and 17% higher antioxidant capacity than control. Individual phenolic compounds, such as caffeic acid, ferulic acid, and kaempferol, exhibited a similar trend to the total phenolic concentration.	[131]
	Winterbor and Redbor kale were harvested in three periods: before frost (>0 °C), after medium (−5.0 °C), and heavy frost (−15.0 °C).	Ascorbic acid (27% and 14%), phenolics (60% and 90%), and antioxidant activity (340% and 80%) were higher after heavy frost in Winterbor and Redbor cultivars, respectively. Anthocyanins also increased significantly for ‘Redbor’ cultivar by 511% and 400%, repectively, with medium and heavy frost.	[132]
	Fifty-day-old kale sprouts (*Brassica oleracea* var. *acephala*) were subjected to low temperature for 4 days: 25 °C/20 °C, 20 °C/15 °C, 15 °C/10 °C	The total carotenoid content was higher at 25 °C/20 °C treatment, but lower at 15 °C/10 °C treatment. The lutein and β-carotene contents decreased 18.3% and 81.6% respectively, by 15℃/10℃ treatment; while zeaxanthin content increased 364% and α-carotene was not affected by cold stress.	[133]
Temperature	Four-day-old kale sprouts were soaked at 50 °C in water for 10, 20, 30, 45, or 60 s.	Heat shock at 50 °C for 20 s induced 150% and 120% higher total phenolic concentration and antioxidants capacity, respectively, than control. Accumulation of total glucosinolates significantly increased by 33% with the same treatment.	[134]
Radiation	Kale sprouts (Half Tall and Black Magic) were radiated with medium light (130 μmol photons/m^2^ s) or in high light (800 μmol photons/m^2^ s).	Total indole glucosinolates decreased in Black Magic cultivar (40%) treated with high light. However, glucoraphanin (150%), glucoerucin (350%), and total aliphatic glucosinolates (175%) content increased. Similarly, Half Tall cultivar showed an increase in glucoberverin (2%), glucoraphanin (400%), glucoerucin (100%), and total aliphatic glucosinolates (66%) with high light treatment.	[135]
	Nine-week-old kale plants (*Brassica oleracea* var. *acephala*) were exposed to UV-B (0–3, 3–6 and 6–9 W/m^2^) for 4 h per day.	The increase in the concentration of total phenolics and flavonoids by UVB treatments was not significantly different.	[136]
	10-day-old kale sprouts (*Brassica oleracea* var*. sabellica*) were light simulated 2 h each day with UV-B (0.0189 W/m^2^) and UV-A (69.502 W/m^2^).	Antioxidant activity increased 2–3-fold in UV-B-treated kale. Kaempferol glycosides decreased in favor of increasing quercetin glycosides. Quercetin-3,7,4 ′-*O*-d-triglucoside, quercetin-3-*O*-caffeoyl-sophoroside-7-*O*-glucoside and quercetin-3-*O*-sin-apoyl-sophoroside-7-*O*-d-glucoside significantly increased 26%, 30% and 33%, respectively, compared to control.	[137]
	5-week-old kale seedlings (*Brassica oleracea* var. *acephala*) were radiated with UV-A LEDs (370 and 385 nm, 30 W/m^2^) continuously for 5 days.	Total phenolic content increased 25% and 42% in kale treated with UV-A LEDs 370 nm and at 385 nm, respectively, at 5 days of treatment. Caffeic acid (200% and 180%) and kaempferol (146% and 168%) concentrations were significantly increased by 370 and 385 nm UV-A radiation, respectively.	[138]

## Data Availability

No new data were created or analyzed in this study. Data sharing is not applicable to this article.

## References

[1] Vidal N.P., Pham H.T., Manful C., Pumphrey R., Nadeem M., Cheema M., Galagedara L., Leke-Aladekoba A., Abbey L., Thomas R. (2018). The Use of Natural Media Amendments to Produce Kale Enhanced with Functional Lipids in Controlled Environment Production System. Sci. Rep..

[2] Šamec D., Urlić B., Salopek-Sondi B. (2019). Kale (*Brassica oleracea var. acephala*) as a Superfood: Review of the Scientific Evidence behind the Statement. Crit. Rev. Food Sci. Nutr..

[3] USDA (2017). Agricultural Research Service: Kale Raw. https://fdc.nal.usda.gov/fdc-app.html#/food-details/323505/nutrients.

[4] Lemos M., Santin J.R., Júnior L.C.K., Niero R., de Andrade S.F. (2011). Gastroprotective Activity of Hydroalcoholic Extract Obtained from the Leaves of *Brassica oleracea var. acephala* DC in Different Animal Models. J. Ethnopharmacol..

[5] Kuerban A., Yaghmoor S.S., Almulaiky Y.Q., Mohamed Y.A., Razvi S.S.I., Hasan M.N., Moselhy S.S., Al-Ghafari A.B., Alsufiani H.M., Kumosani T.A. (2017). Therapeutic Effects of Phytochemicals of Brassicaceae for Management of Obesity. J. Pharm. Res. Int..

[6] Šamec D., Salopek-Sondi B., Nabavi S.M., Silva A.S. (2019). Chapter 3.11—Cruciferous (Brassicaceae) Vegetables. Nonvitamin and Nonmineral Nutritional Supplements.

[7] Abellán Á., Domínguez-Perles R., Moreno D.A., García-Viguera C. (2019). Sorting out the Value of Cruciferous Sprouts as Sources of Bioactive Compounds for Nutrition and Health. Nutrients.

[8] Saini R.K., Keum Y.-S. (2018). Significance of Genetic, Environmental, and Pre- and Postharvest Factors Affecting Carotenoid Contents in Crops: A Review. J. Agric. Food Chem..

[9] Cisneros-Zevallos L. (2003). The Use of Controlled Postharvest Abiotic Stresses as a Tool for Enhancing the Nutraceutical Content and Adding-Value of Fresh Fruits and Vegetables. J. Food Sci..

[10] Jacobo-Velázquez D.A., González-Agüero M., Cisneros-Zevallos L. (2015). Cross-Talk between Signaling Pathways: The Link between Plant Secondary Metabolite Production and Wounding Stress Response. Sci. Rep..

[11] Dudeja P., Gupta R.K., Gupta R.K., Dudeja S.M. (2017). Chapter 40—Nutraceuticals. Food Safety in the 21st Century.

[12] Biegańska-Marecik R., Radziejewska-Kubzdela E., Marecik R. (2017). Characterization of Phenolics, Glucosinolates and Antioxidant Activity of Beverages Based on Apple Juice with Addition of Frozen and Freeze-Dried Curly Kale Leaves (*Brassica oleracea* L. *var. acephala* L.). Food Chem..

[13] Johnson M., McElhenney W.H., Egnin M. (2019). Influence of Green Leafy Vegetables in Diets with an Elevated ω-6:ω-3 Fatty Acid Ratio on Rat Blood Pressure, Plasma Lipids, Antioxidant Status and Markers of Inflammation. Nutrients.

[14] Olsen H., Grimmer S., Aaby K., Saha S., Borge G.I.A. (2012). Antiproliferative Effects of Fresh and Thermal Processed Green and Red Cultivars of Curly Kale (*Brassica oleracea* L. convar. *acephala* var. *sabellica*). J. Agric. Food Chem..

[15] Luang-In V., Saengha W., Buranrat B., Chantiratikul A., Ma N., Ma N. (2020). Cytotoxicity of Selenium-Enriched Chinese Kale (*Brassica oleracea var. alboglabra* L.) Seedlings Against Caco-2, MCF-7 and HepG2 Cancer Cells. Pharmacogn. J..

[16] Sikora E., Bodziarczyk I. (2013). Influence of Diet with Kale on Lipid Peroxides and Malondialdehyde Levels in Blood Serum of Laboratory Rats over Intoxication with Paraquat. Acta Sci. Pol. Technol. Aliment..

[17] Horst M.A., Ong T.P., Jordão A.A., Vannucchi H., Moreno F.S., Lajolo F.M. (2010). Water Extracts of Cabbage and Kale Inhibit Ex Vivo H2O2-Induced DNA Damage but Not Rat Hepatocarcinogenesis. Braz. J. Med. Biol. Res..

[18] Chung E.J., Kim S.Y., Nam Y.J., Park J.H., Hwang H.J., Lee-Kim Y.C. (2005). Effects of Kale Juice Powder on Serum Lipids, Folate and Plasma Homocysteine Levels in Growing Rats. J. Korean Soc. Food Sci. Nutr..

[19] Giacoppo S., Galuppo M., De Nicola G.R., Iori R., Bramanti P., Mazzon E. (2015). Tuscan Black Kale Sprout Extract Bioactivated with Myrosinase: A Novel Natural Product for Neuroprotection by Inflammatory and Oxidative Response during Cerebral Ischemia/Reperfusion Injury in Rat. BMC Complement. Altern. Med..

[20] Lima de Albuquerque C., Comalada M., Camuesco D., Rodríguez-Cabezas M.E., Luiz-Ferreira A., Nieto A., Monteiro de Souza Brito A.R., Zarzuelo A., Gálvez J. (2010). Effect of Kale and Papaya Supplementation in Colitis Induced by Trinitrobenzenesulfonic Acid in the Rat. E-SPEN Eur. E-J. Clin. Nutr. Metab..

[21] Ide T., Suzuki A., Kurokawa M., Minagawa N., Inuzuka H., Ichien G. (2016). Analysis Of Effects Of Kale Juice Consumption Among Subjects With Potential Metabolic Syndrome: A Prospective Single-Arm Clinical Study. J. Hypertens. Cardiol..

[22] Kondo S., Suzuki A., Kurokawa M., Hasumi K. (2016). Intake of Kale Suppresses Postprandial Increases in Plasma Glucose: A Randomized, Double-Blind, Placebo-Controlled, Crossover Study. Biomed. Rep..

[23] Kim S.Y., Yoon S., Kwon S.M., Park K.S., Lee-Kim Y.C. (2008). Kale Juice Improves Coronary Artery Disease Risk Factors in Hypercholesterolemic Men. Biomed. Environ. Sci..

[24] Possenti M., Baima S., Raffo A., Durazzo A., Giusti A.M., Natella F., Mérillon J.-M., Ramawat K.G. (2017). Glucosinolates in Food. Glucosinolates.

[25] Ishida M., Hara M., Fukino N., Kakizaki T., Morimitsu Y. (2014). Glucosinolate Metabolism, Functionality and Breeding for the Improvement of Brassicaceae Vegetables. Breed. Sci..

[26] Narbad A., Rossiter J.T. (2018). Gut Glucosinolate Metabolism and Isothiocyanate Production. Mol. Nutr. Food Res..

[27] Barba F.J., Nikmaram N., Roohinejad S., Khelfa A., Zhu Z., Koubaa M. (2016). Bioavailability of Glucosinolates and Their Breakdown Products: Impact of Processing. Front. Nutr..

[28] Chhajed S., Mostafa I., He Y., Abou-Hashem M., El-Domiaty M., Chen S. (2020). Glucosinolate Biosynthesis and the Glucosinolate–Myrosinase System in Plant Defense. Agronomy.

[29] Keck A.-S., Finley J.W. (2004). Cruciferous Vegetables: Cancer Protective Mechanisms of Glucosinolate Hydrolysis Products and Selenium. Integr. Cancer Ther..

[30] Cartea M.E., Francisco M., Soengas P., Velasco P. (2010). Phenolic Compounds in Brassica Vegetables. Molecules.

[31] Bhandari S.R., Jo J.S., Lee J.G. (2015). Comparison of Glucosinolate Profiles in Different Tissues of Nine Brassica Crops. Molecules.

[32] Sun B., Tian Y.-X., Chen Q., Zhang Y., Luo Y., Wang Y., Li M.-Y., Gong R.-G., Wang X.-R., Zhang F. (2019). Variations in the Glucosinolates of the Individual Edible Parts of Three Stem Mustards (*Brassica Juncea*). R. Soc. Open Sci..

[33] Hahn C., Müller A., Kuhnert N., Albach D. (2016). Diversity of Kale (*Brassica oleracea var. sabellica*): Glucosinolate Content and Phylogenetic Relationships. J. Agric. Food Chem..

[34] Mazumder A., Dwivedi A., du Plessis J. (2016). Sinigrin and Its Therapeutic Benefits. Molecules.

[35] Lopez-Rodriguez N.A., Gaytán-Martínez M., de la Luz Reyes-Vega M., Loarca-Piña G. (2020). Glucosinolates and Isothiocyanates from Moringa Oleifera: Chemical and Biological Approaches. Plant Foods Hum. Nutr. Dordr. Neth..

[36] Dufour V., Stahl M., Baysse C. (2015). The Antibacterial Properties of Isothiocyanates. Microbiol. Read. Engl..

[37] Kołodziejski D., Piekarska A., Hanschen F.S., Pilipczuk T., Tietz F., Kusznierewicz B., Bartoszek A. (2019). Relationship between Conversion Rate of Glucosinolates to Isothiocyanates/Indoles and Genotoxicity of Individual Parts of Brassica Vegetables. Eur. Food Res. Technol..

[38] Yin T.-F., Wang M., Qing Y., Lin Y.-M., Wu D. (2016). Research Progress on Chemopreventive Effects of Phytochemicals on Colorectal Cancer and Their Mechanisms. World J. Gastroenterol..

[39] Mantso T., Anestopoulos I., Lamprianidou E., Kotsianidis I., Pappa A., Panayiotidis M.I. (2019). Isothiocyanate-Induced Cell Cycle Arrest in a Novel In Vitro Exposure Protocol of Human Malignant Melanoma (A375) Cells. Anticancer Res..

[40] Jaafaru M.S., Abd Karim N.A., Mohamed Eliaser E., Maitalata Waziri P., Ahmed H., Mustapha Barau M., Kong L., Abdull Razis A.F. (2018). Nontoxic Glucomoringin-Isothiocyanate (GMG-ITC) Rich Soluble Extract Induces Apoptosis and Inhibits Proliferation of Human Prostate Adenocarcinoma Cells (PC-3). Nutrients.

[41] Lin J., Xu Y., Zhao X., Qiu Z. (2020). Anticancer Activity of Sulforaphane against Human Hepatoblastoma Cells Involves Apoptosis, Autophagy and Inhibition of β-Catenin Signaling Pathway. Arch. Med. Sci..

[42] Serini S., Guarino R., Ottes Vasconcelos R., Celleno L., Calviello G. (2020). The Combination of Sulforaphane and Fernblock^®^ XP Improves Individual Beneficial Effects in Normal and Neoplastic Human Skin Cell Lines. Nutrients.

[43] Georgikou C., Buglioni L., Bremerich M., Roubicek N., Yin L., Gross W., Sticht C., Bolm C., Herr I. (2020). Novel Broccoli Sulforaphane-Based Analogues Inhibit the Progression of Pancreatic Cancer without Side Effects. Biomolecules.

[44] Mitsiogianni M., Trafalis D.T., Franco R., Zoumpourlis V., Pappa A., Panayiotidis M.I. (2021). Sulforaphane and Iberin Are Potent Epigenetic Modulators of Histone Acetylation and Methylation in Malignant Melanoma. Eur. J. Nutr..

[45] Pocasap P., Weerapreeyakul N., Thumanu K. (2019). Alyssin and Iberin in Cruciferous Vegetables Exert Anticancer Activity in HepG2 by Increasing Intracellular Reactive Oxygen Species and Tubulin Depolymerization. Biomol. Ther..

[46] Esteve M. (2020). Mechanisms Underlying Biological Effects of Cruciferous Glucosinolate-Derived Isothiocyanates/Indoles: A Focus on Metabolic Syndrome. Front. Nutr..

[47] Nagata N., Xu L., Kohno S., Ushida Y., Aoki Y., Umeda R., Fuke N., Zhuge F., Ni Y., Nagashimada M. (2017). Glucoraphanin Ameliorates Obesity and Insulin Resistance Through Adipose Tissue Browning and Reduction of Metabolic Endotoxemia in Mice. Diabetes.

[48] Chen J., Bao C., Kim J.T., Cho J.S., Qiu S., Lee H.J. (2018). Sulforaphene Inhibition of Adipogenesis via Hedgehog Signaling in 3T3-L1 Adipocytes. J. Agric. Food Chem..

[49] Chae S.Y., Seo S.G., Yang H., Yu J.G., Suk S.J., Jung E.S., Ji H., Kwon J.Y., Lee H.J., Lee K.W. (2015). Anti-Adipogenic Effect of Erucin in Early Stage of Adipogenesis by Regulating Ras Activity in 3T3-L1 Preadipocytes. J. Funct. Foods.

[50] Yang H., Seo S.G., Shin S.H., Min S., Kang M.J., Yoo R., Kwon J.Y., Yue S., Kim K.H., Cheng J.-X. (2017). 3,3′-Diindolylmethane Suppresses High-Fat Diet-Induced Obesity through Inhibiting Adipogenesis of Pre-Adipocytes by Targeting USP2 Activity. Mol. Nutr. Food Res..

[51] Chang H.-P., Wang M.-L., Hsu C.-Y., Liu M.-E., Chan M.-H., Chen Y.-H. (2011). Suppression of Inflammation-Associated Factors by Indole-3-Carbinol in Mice Fed High-Fat Diets and in Isolated, Co-Cultured Macrophages and Adipocytes. Int. J. Obes..

[52] Yao A., Shen Y., Wang A., Chen S., Zhang H., Chen F., Chen Z., Wei H., Zou Z., Shan Y. (2015). Sulforaphane Induces Apoptosis in Adipocytes via Akt/P70s6k1/Bad Inhibition and ERK Activation. Biochem. Biophys. Res. Commun..

[53] Choi K.-M., Lee Y.-S., Kim W., Kim S.J., Shin K.-O., Yu J.-Y., Lee M.K., Lee Y.-M., Hong J.T., Yun Y.-P. (2014). Sulforaphane Attenuates Obesity by Inhibiting Adipogenesis and Activating the AMPK Pathway in Obese Mice. J. Nutr. Biochem..

[54] Chuang W.-T., Liu Y.-T., Huang C.-S., Lo C.-W., Yao H.-T., Chen H.-W., Lii C.-K. (2019). Benzyl Isothiocyanate and Phenethyl Isothiocyanate Inhibit Adipogenesis and Hepatosteatosis in Mice with Obesity Induced by a High-Fat Diet. J. Agric. Food Chem..

[55] Li C.-X., Gao J.-G., Wan X.-Y., Chen Y., Xu C.-F., Feng Z.-M., Zeng H., Lin Y.-M., Ma H., Xu P. (2019). Allyl Isothiocyanate Ameliorates Lipid Accumulation and Inflammation in Nonalcoholic Fatty Liver Disease via the Sirt1/AMPK and NF-κB Signaling Pathways. World J. Gastroenterol..

[56] Bahadoran Z., Tohidi M., Nazeri P., Mehran M., Azizi F., Mirmiran P. (2012). Effect of Broccoli Sprouts on Insulin Resistance in Type 2 Diabetic Patients: A Randomized Double-Blind Clinical Trial. Int. J. Food Sci. Nutr..

[57] López-Chillón M.T., Carazo-Díaz C., Prieto-Merino D., Zafrilla P., Moreno D.A., Villaño D. (2019). Effects of Long-Term Consumption of Broccoli Sprouts on Inflammatory Markers in Overweight Subjects. Clin. Nutr..

[58] Kikuchi M., Ushida Y., Shiozawa H., Umeda R., Tsuruya K., Aoki Y., Suganuma H., Nishizaki Y. (2015). Sulforaphane-Rich Broccoli Sprout Extract Improves Hepatic Abnormalities in Male Subjects. World J. Gastroenterol..

[59] Shapiro T.A., Fahey J.W., Dinkova-Kostova A.T., Holtzclaw W.D., Stephenson K.K., Wade K.L., Ye L., Talalay P. (2006). Safety, Tolerance, and Metabolism of Broccoli Sprout Glucosinolates and Isothiocyanates: A Clinical Phase I Study. Nutr. Cancer.

[60] Yagi M., Nakatsuji Y., Maeda A., Ota H., Kamikubo R., Miyoshi N., Nakamura Y., Akagawa M. (2018). Phenethyl Isothiocyanate Activates Leptin Signaling and Decreases Food Intake. PLoS ONE.

[61] Jayakumar P., Pugalendi K.V., Sankaran M. (2014). Attenuation of Hyperglycemia-Mediated Oxidative Stress by Indole-3-Carbinol and Its Metabolite 3,3′- Diindolylmethane in C57BL/6J Mice. J. Physiol. Biochem..

[62] Chinni S.R., Sarkar F.H. (2002). Akt Inactivation Is a Key Event in Indole-3-Carbinol-Induced Apoptosis in PC-3 Cells. Clin. Cancer Res..

[63] Chen D.Z., Qi M., Auborn K.J., Carter T.H. (2001). Indole-3-Carbinol and Diindolylmethane Induce Apoptosis of Human Cervical Cancer Cells and in Murine HPV16-Transgenic Preneoplastic Cervical Epithelium. J. Nutr..

[64] Choi Y., Kim Y., Park S., Lee K.W., Park T. (2012). Indole-3-Carbinol Prevents Diet-Induced Obesity through Modulation of Multiple Genes Related to Adipogenesis, Thermogenesis or Inflammation in the Visceral Adipose Tissue of Mice. J. Nutr. Biochem..

[65] Meng Q., Yuan F., Goldberg I.D., Rosen E.M., Auborn K., Fan S. (2000). Indole-3-Carbinol Is a Negative Regulator of Estrogen Receptor-Alpha Signaling in Human Tumor Cells. J. Nutr..

[66] Garikapaty V.P.S., Ashok B.T., Chen Y.G., Mittelman A., Iatropoulos M., Tiwari R.K. (2005). Anti-Carcinogenic and Anti-Metastatic Properties of Indole-3-Carbinol in Prostate Cancer. Oncol. Rep..

[67] Ayad R., Akkal S., Atta-ur-Rahman (2019). Chapter 12—Phytochemistry and Biological Activities of Algerian Centaurea and Related Genera. Studies in Natural Products Chemistry.

[68] Lu L., Xu L., Guo Y., Zhang D., Qi T., Jin L., Gu G., Xu L., Xiao M. (2015). Glycosylation of Phenolic Compounds by the Site-Mutated β-Galactosidase from Lactobacillus Bulgaricus L3. PLoS ONE.

[69] Hussain M.B., Hassan S., Waheed M., Javed A., Farooq M.A., Tahir A. (2019). Bioavailability and Metabolic Pathway of Phenolic Compounds.

[70] Botelho G., Canas S., Lameiras J., Grumezescu A.M. (2017). 14—Development of Phenolic Compounds Encapsulation Techniques as a Major Challenge for Food Industry and for Health and Nutrition Fields. Nutrient Delivery.

[71] Akdaş Z.Z., Bakkalbaşı E. (2017). Influence of Different Cooking Methods on Color, Bioactive Compounds, and Antioxidant Activity of Kale. Int. J. Food Prop..

[72] Wang Y.-Q., Hu L.-P., Liu G.-M., Zhang D.-S., He H.-J. (2017). Evaluation of the Nutritional Quality of Chinese Kale (*Brassica alboglabra* bailey) Using UHPLC-Quadrupole-Orbitrap MS/MS-Based Metabolomics. Molecules.

[73] Jeon J., Kim J.K., Kim H., Kim Y.J., Park Y.J., Kim S.J., Kim C., Park S.U. (2018). Transcriptome Analysis and Metabolic Profiling of Green and Red Kale (*Brassica oleracea* var. *acephala*) Seedlings. Food Chem..

[74] Ferioli F., Giambanelli E., D’Antuono L.F., Costa H.S., Albuquerque T.G., Silva A.S., Hayran O., Koçaoglu B. (2013). Comparison of Leafy Kale Populations from Italy, Portugal, and Turkey for Their Bioactive Compound Content: Phenolics, Glucosinolates, Carotenoids, and Chlorophylls. J. Sci. Food Agric..

[75] Serçe A., Toptancı B.Ç., Tanrıkut S.E., Altaş S., Kızıl G., Kızıl S., Kızıl M. (2016). Assessment of the Antioxidant Activity of Silybum Marianum Seed Extract and Its Protective Effect against DNA Oxidation, Protein Damage and Lipid Peroxidation. Food Technol. Biotechnol..

[76] Yang H., Kang M.J., Hur G., Lee T.K., Park I.S., Seo S.G., Yu J.G., Song Y.S., Park J.H.Y., Lee K.W. (2020). Sulforaphene Suppresses Adipocyte Differentiation via Induction of Post-Translational Degradation of CCAAT/Enhancer Binding Protein Beta (C/EBPβ). Nutrients.

[77] Ha H.J., Kwon Y.S., Park S.M., Shin T., Park J.H., Kim H.C., Kwon M.S., Wie M.B. (2003). Quercetin Attenuates Oxygen-Glucose Deprivation- and Excitotoxin-Induced Neurotoxicity in Primary Cortical Cell Cultures. Biol. Pharm. Bull..

[78] Perez A., Gonzalez-Manzano S., Jimenez R., Perez-Abud R., Haro J.M., Osuna A., Santos-Buelga C., Duarte J., Perez-Vizcaino F. (2014). The Flavonoid Quercetin Induces Acute Vasodilator Effects in Healthy Volunteers: Correlation with Beta-Glucuronidase Activity. Pharmacol. Res..

[79] Hämäläinen M., Nieminen R., Asmawi M.Z., Vuorela P., Vapaatalo H., Moilanen E. (2011). Effects of Flavonoids on Prostaglandin E2 Production and on COX-2 and MPGES-1 Expressions in Activated Macrophages. Planta Med..

[80] Zhang Z., Chen S., Mei H., Xuan J., Guo X., Couch L., Dobrovolsky V.N., Guo L., Mei N. (2015). Ginkgo Biloba Leaf Extract Induces DNA Damage by Inhibiting Topoisomerase II Activity in Human Hepatic Cells. Sci. Rep..

[81] Shimojo Y., Ozawa Y., Toda T., Igami K., Shimizu T. (2018). Probiotic Lactobacillus Paracasei A221 Improves the Functionality and Bioavailability of Kaempferol-Glucoside in Kale by Its Glucosidase Activity. Sci. Rep..

[82] Ayaz F.A., Hayırlıoglu-Ayaz S., Alpay-Karaoglu S., Grúz J., Valentová K., Ulrichová J., Strnad M. (2008). Phenolic Acid Contents of Kale (*Brassica oleraceae* L. var. *acephala* DC.) Extracts and Their Antioxidant and Antibacterial Activities. Food Chem..

[83] Cuevas E., Limón D., Pérez-Severiano F., Díaz A., Ortega L., Zenteno E., Guevara J. (2009). Antioxidant Effects of Epicatechin on the Hippocampal Toxicity Caused by Amyloid-Beta 25-35 in Rats. Eur. J. Pharmacol..

[84] Li Y.-L., Guo H., Zhao Y.-Q., Li A.-F., Ren Y.-Q., Zhang J.-W. (2017). Quercetin Protects Neuronal Cells from Oxidative Stress and Cognitive Degradation Induced by Amyloid β-Peptide Treatment. Mol. Med. Rep..

[85] Rubio-Perez J.M., Morillas-Ruiz J.M. (2012). A Review: Inflammatory Process in Alzheimer’s Disease, Role of Cytokines. Sci. World J..

[86] Panche A.N., Diwan A.D., Chandra S.R. (2016). Flavonoids: An Overview. J. Nutr. Sci..

[87] Lozano-Sepúlveda S.A., Rincón-Sanchez A.R., Rivas-Estilla A.M. (2019). Antioxidants Benefits in Hepatitis C Infection in the New DAAs Era. Ann. Hepatol..

[88] Rufino A.T., Costa V.M., Carvalho F., Fernandes E. (2021). Flavonoids as Antiobesity Agents: A Review. Med. Res. Rev..

[89] Steffen Y., Gruber C., Schewe T., Sies H. (2008). Mono-O-Methylated Flavanols and Other Flavonoids as Inhibitors of Endothelial NADPH Oxidase. Arch. Biochem. Biophys..

[90] Nićiforović N., Abramovič H. (2014). Sinapic Acid and Its Derivatives: Natural Sources and Bioactivity. Compr. Rev. Food Sci. Food Saf..

[91] Maoka T. (2020). Carotenoids as Natural Functional Pigments. J. Nat. Med..

[92] Canene-Adams K., Erdman J.W., Britton G., Pfander H., Liaaen-Jensen S. (2009). Absorption, Transport, Distribution in Tissues and Bioavailability. Carotenoids: Volume 5: Nutrition and Health.

[93] Wingerath T., Sies H., Stahl W. (1998). Xanthophyll Esters in Human Skin. Arch. Biochem. Biophys..

[94] Muzhingi T., Yeum K.-J., Bermudez O., Tang G., Siwela A.H. (2017). Peanut Butter Increases the Bioavailability and Bioconversion of Kale β-Carotene to Vitamin A. Asia Pac. J. Clin. Nutr..

[95] Becerra-Moreno A., Alanís-Garza P.A., Mora-Nieves J.L., Mora-Mora J.P., Jacobo-Velázquez D.A. (2014). Kale: An Excellent Source of Vitamin C, pro-Vitamin A, Lutein and Glucosinolates. CyTA J. Food.

[96] Lefsrud M., Kopsell D., Wenzel A., Sheehan J. (2007). Changes in Kale (*Brassica oleracea* L. *var. acephala*) Carotenoid and Chlorophyll Pigment Concentrations during Leaf Ontogeny. Sci. Hortic..

[97] Kim M.J., Chiu Y.-C., Ku K.-M. (2017). Glucosinolates, Carotenoids, and Vitamins E and K Variation from Selected Kale and Collard Cultivars. J. Food Qual..

[98] Rafi M.M., Kanakasabai S., Gokarn S.V., Krueger E.G., Bright J.J. (2015). Dietary Lutein Modulates Growth and Survival Genes in Prostate Cancer Cells. J. Med. Food.

[99] Ma L., Lin X.-M., Zou Z.-Y., Xu X.-R., Li Y., Xu R. (2009). A 12-Week Lutein Supplementation Improves Visual Function in Chinese People with Long-Term Computer Display Light Exposure. Br. J. Nutr..

[100] Sun Y.-X., Liu T., Dai X.-L., Zheng Q.-S., Hui B.-D., Jiang Z.-F. (2014). Treatment with Lutein Provides Neuroprotection in Mice Subjected to Transient Cerebral Ischemia. J. Asian Nat. Prod. Res..

[101] Binawade Y., Jagtap A. (2013). Neuroprotective Effect of Lutein against 3-Nitropropionic Acid-Induced Huntington’s Disease-like Symptoms: Possible Behavioral, Biochemical, and Cellular Alterations. J. Med. Food.

[102] Qiu X., Gao D.-H., Xiang X., Xiong Y.-F., Zhu T.-S., Liu L.-G., Sun X.-F., Hao L.-P. (2015). Ameliorative Effects of Lutein on Non-Alcoholic Fatty Liver Disease in Rats. World J. Gastroenterol..

[103] Sowmya Shree G., Yogendra Prasad K., Arpitha H.S., Deepika U.R., Nawneet Kumar K., Mondal P., Ganesan P. (2017). β-Carotene at Physiologically Attainable Concentration Induces Apoptosis and down-Regulates Cell Survival and Antioxidant Markers in Human Breast Cancer (MCF-7) Cells. Mol. Cell. Biochem..

[104] Levy Y., Zaltsberg H., Ben-Amotz A., Kanter Y., Aviram M. (2000). Dietary Supplementation of a Natural Isomer Mixture of Beta-Carotene Inhibits Oxidation of LDL Derived from Patients with Diabetes Mellitus. Ann. Nutr. Metab..

[105] Boccardi V., Arosio B., Cari L., Bastiani P., Scamosci M., Casati M., Ferri E., Bertagnoli L., Ciccone S., Rossi P.D. (2020). Beta-Carotene, Telomerase Activity and Alzheimer’s Disease in Old Age Subjects. Eur. J. Nutr..

[106] Arnold C., Jentsch S., Dawczynski J., Böhm V. (2013). Age-Related Macular Degeneration: Effects of a Short-Term Intervention with an Oleaginous Kale Extract—A Pilot Study. Nutrition.

[107] Shi J., Ho C.-T., Shahidi F. (2010). Functional Foods of the East.

[108] Pagliaro B., Santolamazza C., Simonelli F., Rubattu S. (2015). Phytochemical Compounds and Protection from Cardiovascular Diseases: A State of the Art. BioMed. Res. Int..

[109] Ranard K.M., Jeon S., Mohn E.S., Griffiths J.C., Johnson E.J., Erdman J.W. (2017). Dietary Guidance for Lutein: Consideration for Intake Recommendations Is Scientifically Supported. Eur. J. Nutr..

[110] Cisneros-Zevallos L., Jacobo-Velázquez D.A., Pech J.C., Koiwa H., Pessarakli M. (2014). Signaling Molecules Involved in the Postharvest Stress Response of Plants: Quality Changes and Synthesis of Secondary Metabolites. Handbook of Plant and Crop Physiology.

[111] Cao M.-J., Wang Z., Zhao Q., Mao J.-L., Speiser A., Wirtz M., Hell R., Zhu J.-K., Xiang C.-B. (2014). Sulfate Availability Affects ABA Levels and Germination Response to ABA and Salt Stress in Arabidopsis Thaliana. Plant J. Cell Mol. Biol..

[112] Jacobo-Velázquez D.A., Cisneros-Zevallos L. (2012). An Alternative Use of Horticultural Crops: Stressed Plants as Biofactories of Bioactive Phenolic Compounds. Agriculture.

[113] Cisneros-Zevallos L., Jacobo-Velázquez D.A. (2020). Controlled Abiotic Stresses Revisited: From Homeostasis through Hormesis to Extreme Stresses and the Impact on Nutraceuticals and Quality during Pre- and Postharvest Applications in Horticultural Crops. J. Agric. Food Chem..

[114] Hideg É., Jansen M.A.K., Strid Å. (2013). UV-B Exposure, ROS, and Stress: Inseparable Companions or Loosely Linked Associates?. Trends Plant Sci..

[115] Carillo P., Soteriou G.A., Kyriacou M.C., Giordano M., Raimondi G., Napolitano F., Di Stasio E., Mola I.D., Mori M., Rouphael Y. (2021). Regulated Salinity Eustress in a Floating Hydroponic Module of Sequentially Harvested Lettuce Modulates Phytochemical Constitution, Plant Resilience, and Post-Harvest Nutraceutical Quality. Agronomy.

[116] Vargas-Hernandez M., Macias-Bobadilla I., Guevara-Gonzalez R.G., Romero-Gomez S.D.J., Rico-Garcia E., Ocampo-Velazquez R.V., Alvarez-Arquieta L.d.L., Torres-Pacheco I. (2017). Plant Hormesis Management with Biostimulants of Biotic Origin in Agriculture. Front. Plant Sci..

[117] Rouphael Y., Kyriacou M.C., Carillo P., Pizzolongo F., Romano R., Sifola M.I. (2019). Chemical Eustress Elicits Tailored Responses and Enhances the Functional Quality of Novel Food Perilla Frutescens. Molecules.

[118] Pandey N., Iqbal Z., Pandey B.K., Sawant S.V. (2017). Phytohormones and Drought Stress: Plant Responses to Transcriptional Regulation. Mechanism of Plant Hormone Signaling under Stress.

[119] Hirani A.H., Li G., Zelmer C.D., McVetty P.B.E., Asif M., Goyal A. (2012). Molecular Genetics of Glucosinolate Biosynthesis in Brassicas: Genetic Manipulation and Application Aspects.

[120] Groenbaek M., Jensen S., Neugart S., Schreiner M., Kidmose U., Kristensen H.L. (2016). Nitrogen Split Dose Fertilization, Plant Age and Frost Effects on Phytochemical Content and Sensory Properties of Curly Kale (*Brassica oleracea* L. var. *sabellica*). Food Chem..

[121] Park Y.-J., Lee H.-M., Shin M., Arasu M.V., Chung D.Y., Al-Dhabi N.A., Kim S.-J. (2018). Effect of Different Proportion of Sulphur Treatments on the Contents of Glucosinolate in Kale (*Brassica oleracea var. acephala*) Commonly Consumed in Republic of Korea. Saudi J. Biol. Sci..

[122] Kopsell D.E., Kopsell D.A., Randle W.M., Coolong T.W., Sams C.E., Curran-Celentano J. (2003). Kale Carotenoids Remain Stable While Flavor Compounds Respond to Changes in Sulfur Fertility. J. Agric. Food Chem..

[123] Kim S.Y., Park J.-E., Kim E.O., Lim S.J., Nam E.J., Yun J.H., Yoo G., Oh S.-R., Kim H.S., Nho C.W. (2018). Exposure of Kale Root to NaCl and Na_2_SeO_3_ Increases Isothiocyanate Levels and Nrf_2_ Signalling without Reducing Plant Root Growth. Sci. Rep..

[124] Wang M., Cai C., Lin J., Tao H., Zeng W., Zhang F., Miao H., Sun B., Wang Q. (2020). Combined Treatment of Epi-Brassinolide and NaCl Enhances the Main Phytochemicals in Chinese Kale Sprouts. Food Chem..

[125] Linić I., Šamec D., Grúz J., Vujčić Bok V., Strnad M., Salopek-Sondi B. (2019). Involvement of Phenolic Acids in Short-Term Adaptation to Salinity Stress Is Species-Specific among Brassicaceae. Plants.

[126] Sun B., Yan H., Zhang F., Wang Q. (2012). Effects of Plant Hormones on Main Health-Promoting Compounds and Antioxidant Capacity of Chinese Kale. Food Res. Int..

[127] Ku K.M., Juvik J.A. (2013). Environmental Stress and Methyl Jasmonate-Mediated Changes in Flavonoid Concentrations and Antioxidant Activity in Broccoli Florets and Kale Leaf Tissues. HortScience.

[128] Yi G.-E., Robin A.H.K., Yang K., Park J.-I., Hwang B.H., Nou I.-S. (2016). Exogenous Methyl Jasmonate and Salicylic Acid Induce Subspecies-Specific Patterns of Glucosinolate Accumulation and Gene Expression in *Brassica oleracea* L.. Molecules.

[129] Barickman T.C., Ku K.-M., Sams C.E. (2020). Differing Precision Irrigation Thresholds for Kale (*Brassica oleracea* L. var. *acephala*) Induces Changes in Physiological Performance, Metabolites, and Yield. Environ. Exp. Bot..

[130] Yoon H.I., Zhang W., Son J.E. (2020). Optimal Duration of Drought Stress Near Harvest for Promoting Bioactive Compounds and Antioxidant Capacity in Kale with or without UV-B Radiation in Plant Factories. Plants.

[131] Lee J.-H., Oh M.-M. (2015). Short-Term Low Temperature Increases Phenolic Antioxidant Levels in Kale. Hortic. Environ. Biotechnol..

[132] Jurkow R., Wurst A., Kalisz A., Sękara A., Cebula S. (2019). Cold Stress Modifies Bioactive Compounds of Kale Cultivars during Fall–Winter Harvests. Acta Agrobot..

[133] Hwang S.-J., Chun J.-H., Kim S.-J. (2017). Effect of Cold Stress on Carotenoids in Kale Leaves (*Brassica oleracea*). Korean J. Environ. Agric..

[134] Lee M.-J., Lim S., Kim J., Oh M.-M. (2012). Heat Shock Treatments Induce the Accumulation of Phytochemicals in Kale Sprouts. Hortic. Sci. Technol..

[135] Alegre S., Pascual J., Trotta A., Gollan P.J., Yang W., Yang B., Aro E.-M., Burow M., Kangasjärvi S. (2019). Growth under High Light and Elevated Temperature Affects Metabolic Responses and Accumulation of Health-Promoting Metabolites in Kale Varieties. bioRxiv.

[136] Yoon H.I., Kim D., Son J.E. (2020). Spatial and Temporal Bioactive Compound Contents and Chlorophyll Fluorescence of Kale (*Brassica oleracea* L.) Under UV-B Exposure Near Harvest Time in Controlled Environments. Photochem. Photobiol..

[137] Klopsch R., Baldermann S., Voss A., Rohn S., Schreiner M., Neugart S. (2019). Narrow-Banded UVB Affects the Stability of Secondary Plant Metabolites in Kale (*Brassica oleracea* var. *sabellica*) and Pea (*Pisum Sativum*) Leaves Being Added to Lentil Flour Fortified Bread: A Novel Approach for Producing Functional Foods. Foods.

[138] Schreiner M., Krumbein A., Mewis I., Ulrichs C., Huyskens-Keil S. (2009). Short-Term UV-B Radiation Effects on Secondary Metabolism in Different Organs of *Tropaeolum majus* L.. Innov. Food Sci. Emerg. Technol..

[139] Jaganjac M., Milkovic L., Sunjic S.B., Zarkovic N. (2020). The NRF2, Thioredoxin, and Glutathione System in Tumorigenesis and Anticancer Therapies. Antioxidants.

[140] Brodowska M.S., Kurzyna-Szklarek M., Haliniarz M. (2016). Selenium in the environment. J. Elem..

[141] Guardado-Félix D., Antunes-Ricardo M., Rocha-Pizaña M.R., Martínez-Torres A.-C., Gutiérrez-Uribe J.A., Serna Saldivar S.O. (2019). Chickpea (*Cicer arietinum* L.) Sprouts Containing Supranutritional Levels of Selenium Decrease Tumor Growth of Colon Cancer Cells Xenografted in Immune-Suppressed Mice. J. Funct. Foods.

[142] Cox D.N., Bastiaans K. (2007). Understanding Australian Consumers’ Perceptions of Selenium and Motivations to Consume Selenium Enriched Foods. Food Qual. Prefer..

[143] Schiavon M., Pilon-Smits E.A.H. (2017). The Fascinating Facets of Plant Selenium Accumulation—Biochemistry, Physiology, Evolution and Ecology. New Phytol..

[144] Guardado-Félix D., Serna-Saldivar S.O., Cuevas-Rodríguez E.O., Jacobo-Velázquez D.A., Gutiérrez-Uribe J.A. (2017). Effect of Sodium Selenite on Isoflavonoid Contents and Antioxidant Capacity of Chickpea (*Cicer arietinum* L.) Sprouts. Food Chem..

[145] Feng R., Wei C., Tu S. (2013). The Roles of Selenium in Protecting Plants against Abiotic Stresses. Environ. Exp. Bot..

[146] Brosché M., Strid Å. (2003). Molecular Events Following Perception of Ultraviolet-B Radiation by Plants. Physiol. Plant..

[147] Barickman T.C., Kopsell D.A., Sams C.E. (2013). Selenium Influences Glucosinolate and Isothiocyanates and Increases Sulfur Uptake in Arabidopsis Thaliana and Rapid-Cycling *Brassica oleracea*. J. Agric. Food Chem..

[148] Bartels D., Sunkar R. (2005). Drought and Salt Tolerance in Plants. Crit. Rev. Plant Sci..

[149] Chaman M.E. (2008). Variaciones en el Contenido Relativo de Agua y la Concentración de Prolina en Capsicum annuum L. Inducido por NaCl. Tesis de Doctorado en Ciencias Biológicas.

[150] Sudhakar C., Lakshmi A., Giridarakumar S. (2001). Changes in the Antioxidant Enzyme Efficacy in Two High Yielding Genotypes of Mulberry (*Morus alba* L.) under NaCl Salinity. Plant Sci..

[151] Dat J., Vandenabeele S., Vranová E., Van Montagu M., Inzé D., Van Breusegem F. (2000). Dual Action of the Active Oxygen Species during Plant Stress Responses. Cell. Mol. Life Sci..

[152] Hasegawa P.M., Bressan R.A., Zhu J.-K., Bohnert H.J. (2000). Plant Cellular and Molecular Responses to High Salinity. Annu. Rev. Plant Physiol. Plant Mol. Biol..

[153] Zhao J., Davis L.C., Verpoorte R. (2005). Elicitor Signal Transduction Leading to Production of Plant Secondary Metabolites. Biotechnol. Adv..

[154] Park W.T., Kim Y.B., Seo J.M., Kim S.-J., Chung E., Lee J.-H., Park S.U. (2013). Accumulation of Anthocyanin and Associated Gene Expression in Radish Sprouts Exposed to Light and Methyl Jasmonate. J. Agric. Food Chem..

[155] Augustine R., Bisht N.C., Mérillon J.-M., Ramawat K.G. (2017). Regulation of Glucosinolate Metabolism: From Model Plant Arabidopsis Thaliana to Brassica Crops. Glucosinolates.

[156] Skirycz A., Reichelt M., Burow M., Birkemeyer C., Rolcik J., Kopka J., Zanor M.I., Gershenzon J., Strnad M., Szopa J. (2006). DOF Transcription Factor AtDof1.1 (OBP2) Is Part of a Regulatory Network Controlling Glucosinolate Biosynthesis in Arabidopsis. Plant J. Cell Mol. Biol..

[157] Mikkelsen M.D., Petersen B.L., Glawischnig E., Jensen A.B., Andreasson E., Halkier B.A. (2003). Modulation of CYP79 Genes and Glucosinolate Profiles in Arabidopsis by Defense Signaling Pathways. Plant Physiol..

[158] Baskar V., Gururani M.A., Yu J.W., Park S.W. (2012). Engineering Glucosinolates in Plants: Current Knowledge and Potential Uses. Appl. Biochem. Biotechnol..

[159] Chiu Y.-C., Juvik J.A., Ku K.-M. (2018). Targeted Metabolomic and Transcriptomic Analyses of “Red Russian” Kale (*Brassicae napus var. pabularia*) Following Methyl Jasmonate Treatment and Larval Infestation by the Cabbage Looper (Trichoplusia Ni Hübner). Int. J. Mol. Sci..

[160] Cruz de Carvalho M.H. (2008). Drought Stress and Reactive Oxygen Species: Production, Scavenging and Signaling. Plant Signal. Behav..

[161] Claeys H., Inzé D. (2013). The Agony of Choice: How Plants Balance Growth and Survival under Water-Limiting Conditions. Plant Physiol..

[162] Fischer B.B., Hideg É., Krieger-Liszkay A. (2013). Production, Detection, and Signaling of Singlet Oxygen in Photosynthetic Organisms. Antioxid. Redox Signal..

[163] Nelson N., Junge W. (2015). Structure and Energy Transfer in Photosystems of Oxygenic Photosynthesis. Annu. Rev. Biochem..

[164] Issarakraisila M., Ma Q., Turner D.W. (2007). Photosynthetic and Growth Responses of Juvenile Chinese Kale (*Brassica oleracea* var. *alboglabra*) and Caisin (*Brassica rapa* subsp. *parachinensis*) to Waterlogging and Water Deficit. Sci. Hortic..

[165] Zhu Z., Sun B., Xu X., Chen H., Zou L., Chen G., Cao B., Chen C., Lei J. (2016). Overexpression of AtEDT1/HDG11 in Chinese Kale (*Brassica oleracea* var. *alboglabra*) Enhances Drought and Osmotic Stress Tolerance. Front. Plant Sci..

[166] Chaves-Barrantes N.F., Gutiérrez-Soto M.V. (2017). Respuestas al estrés por calor en los cultivos. I. aspectos moleculares, bioquímicos y fisiológicos. Agron. Mesoam..

[167] Wahid A., Gelani S., Ashraf M., Foolad M.R. (2007). Heat Tolerance in Plants: An Overview. Environ. Exp. Bot..

[168] Wang L.-C., Tsai M.-C., Chang K.-Y., Fan Y.-S., Yeh C.-H., Wu S.-J. (2011). Involvement of the Arabidopsis HIT1/AtVPS53 Tethering Protein Homologue in the Acclimation of the Plasma Membrane to Heat Stress. J. Exp. Bot..

[169] Savchenko G.E., Klyuchareva E.A., Abramchik L.M., Serdyuchenko E.V. (2002). Effect of Periodic Heat Shock on the Inner Membrane System of Etioplasts. Russ. J. Plant Physiol..

[170] Los D.A., Murata N. (2004). Membrane Fluidity and Its Roles in the Perception of Environmental Signals. Biochim. Biophys. Acta BBA-Biomembr..

[171] Porch T.G., Hall A.E., Kole C. (2013). Heat Tolerance. Genomics and Breeding for Climate-Resilient Crops: Vol. 2 Target Traits.

[172] Almeselmani M., Deshmukh P.S., Sairam R.K., Kushwaha S.R., Singh T.P. (2006). Protective Role of Antioxidant Enzymes under High Temperature Stress. Plant Sci..

[173] Nagesh Babu R., Rangaiah D.V. (2008). High Temperature and Salt Stress Response in French Bean (*Phaseolus vulgaris*). Aust. J. Crop Sci..

[174] Upchurch R.G. (2008). Fatty Acid Unsaturation, Mobilization, and Regulation in the Response of Plants to Stress. Biotechnol. Lett..

[175] Hikosaka K., Ishikawa K., Borjigidai A., Muller O., Onoda Y. (2006). Temperature Acclimation of Photosynthesis: Mechanisms Involved in the Changes in Temperature Dependence of Photosynthetic Rate. J. Exp. Bot..

[176] Hasanuzzaman M., Nahar K., Alam M.M., Roychowdhury R., Fujita M. (2013). Physiological, Biochemical, and Molecular Mechanisms of Heat Stress Tolerance in Plants. Int. J. Mol. Sci..

[177] Gill S.S., Tuteja N. (2010). Reactive Oxygen Species and Antioxidant Machinery in Abiotic Stress Tolerance in Crop Plants. Plant Physiol. Biochem..

[178] Müller-Xing R., Xing Q., Goodrich J. (2014). Footprints of the Sun: Memory of UV and Light Stress in Plants. Front. Plant Sci..

[179] Jenkins G.I. (2009). Signal Transduction in Responses to UV-B Radiation. Annu. Rev. Plant Biol..

[180] Jenkins G.I., Fuglevand G., Christie J.M., Lumsden P. (2010). UV-B Perception and Signal Transduction. Plants and UV-B: Responses to Environmental Change.

[181] Brown B.A., Cloix C., Jiang G.H., Kaiserli E., Herzyk P., Kliebenstein D.J., Jenkins G.I. (2005). A UV-B-Specific Signaling Component Orchestrates Plant UV Protection. Proc. Natl. Acad. Sci. USA.

[182] Christie J.M., Arvai A.S., Baxter K.J., Heilmann M., Pratt A.J., O’Hara A., Kelly S.M., Hothorn M., Smith B.O., Hitomi K. (2012). Plant UVR8 Photoreceptor Senses UV-B by Tryptophan-Mediated Disruption of Cross-Dimer Salt Bridges. Science.

[183] Favory J.-J., Stec A., Gruber H., Rizzini L., Oravecz A., Funk M., Albert A., Cloix C., Jenkins G.I., Oakeley E.J. (2009). Interaction of COP1 and UVR8 Regulates UV-B-Induced Photomorphogenesis and Stress Acclimation in Arabidopsis. EMBO J..

[184] Kaiserli E., Jenkins G.I. (2007). UV-B Promotes Rapid Nuclear Translocation of the Arabidopsis UV-B Specific Signaling Component UVR8 and Activates Its Function in the Nucleus. Plant Cell.

[185] Tilbrook K., Arongaus A.B., Binkert M., Heijde M., Yin R., Ulm R. (2013). The UVR8 UV-B Photoreceptor: Perception, Signaling and Response. Arab. Book.

[186] Verdaguer D., Jansen M.A.K., Llorens L., Morales L.O., Neugart S. (2017). UV-A Radiation Effects on Higher Plants: Exploring the Known Unknown. Plant Sci..

[187] Kuhlmann F., Müller C. (2009). Independent Responses to Ultraviolet Radiation and Herbivore Attack in Broccoli. J. Exp. Bot..

[188] Mewis I., Schreiner M., Nguyen C.N., Krumbein A., Ulrichs C., Lohse M., Zrenner R. (2012). UV-B Irradiation Changes Specifically the Secondary Metabolite Profile in Broccoli Sprouts: Induced Signaling Overlaps with Defense Response to Biotic Stressors. Plant Cell Physiol..

[189] Srivastava A.K., Suresh Kumar J., Suprasanna P. (2021). Seed ‘Primeomics’: Plants Memorize Their Germination under Stress. Biol. Rev..

[190] Garfin G., Franco G., Blanco H., Comrie A., Gonzalez P., Piechota T., Smyth R., Waskom R., Melillo J.M., Richmond T. (2014). Chapter 20: Southwest. Climate Change Impacts in the United States: The Third National Climate Assessment.

[191] Satheesh N., Workneh Fanta S. (2020). Kale: Review on Nutritional Composition, Bio-Active Compounds, Anti-Nutritional Factors, Health Beneficial Properties and Value-Added Products. Cogent. Food Agric..

[192] Jacobo-Velázquez D.A., Benavides J. (2021). Non-Thermal Technologies as Tools to Increase the Content of Health-Promoting Compounds in Whole Fruits and Vegetables while Retaining Quality Attributes. Foods.

